# Comparison of T790M Acquisition After Treatment With First- and Second-Generation Tyrosine-Kinase Inhibitors: A Systematic Review and Network Meta-Analysis

**DOI:** 10.3389/fonc.2022.869390

**Published:** 2022-06-28

**Authors:** Po-Chun Hsieh, Yao-Kuang Wu, Chun-Yao Huang, Mei-Chen Yang, Chan-Yen Kuo, I-Shiang Tzeng, Chou-Chin Lan

**Affiliations:** ^1^ Department of Chinese Medicine, Taipei Tzu Chi Hospital, Buddhist Tzu Chi Medical Foundation, New Taipei City, Taiwan; ^2^ Division of Pulmonary Medicine, Taipei Tzu Chi Hospital, Buddhist Tzu Chi Medical Foundation, New Taipei City, Taiwan; ^3^ School of Medicine, Tzu-Chi University, Hualien, Taiwan; ^4^ Department of Research, Taipei Tzu Chi Hospital, Buddhist Tzu Chi Medical Foundation, New Taipei City, Taiwan

**Keywords:** non-small cell lung cancer, adenocarcinoma, epidermal growth factor receptor, tyrosine kinase inhibitors, T790M acquisition

## Abstract

**Background:**

Lung adenocarcinoma is a common disease with a high mortality rate. Epidermal growth factor receptor (*EGFR*) mutations are found in adenocarcinomas, and oral EGFR-tyrosine kinase inhibitors (EGFR-TKIs) show good responses. EGFR-TKI therapy eventually results in resistance, with the most common being T790M. T790M is also a biomarker for predicting resistance to first- and second-generation EGFR-TKIs and is sensitive to osimertinib. The prognosis was better for patients with acquired T790M who were treated with osimertinib than for those treated with chemotherapy. Therefore, T790M mutation is important for deciding further treatment and prognosis. Previous studies based on small sample sizes have reported very different T790 mutation rates. We conducted a meta-analysis to evaluate the T790M mutation rate after EGFR-TKI treatment.

**Methods:**

We systematic reviewed the electronic databases to evaluate the T790M mutation rate after treatment with first-generation (gefitinib, erlotinib, and icotinib) and second-generation (afatinib and dacomitinib) EGFR-TKIs. Random-effects network meta-analysis and single-arm meta-analysis were conducted to estimate the T790M mutation rate of the target EGFR-TKIs.

**Results:**

A total of 518 studies were identified, of which 29 were included. Compared with afatinib, a higher odds ratio (OR) of the T790M mutation rate was observed after erlotinib [OR = 1.48; 95% confidence interval (CI):1.09–2.00] and gefitinib (OR = 1.45; 95% CI: 1.11–1.90) treatments. An even OR of the T790M mutation rate was noted after icotinib treatment (OR = 0.91, 95% CI: 0.46–1.79) compared with that after afatinib. The T790M mutation rate was significantly lower with afatinib (33%) than that with gefitinib (49%) and erlotinib treatments (47%) (*p* < 0.001). The acquired T790M mutation rate in all participants was slightly lower in Asians (43%) than that in Caucasians (47%).

**Conclusions:**

Erlotinib and gefitinib had a higher OR for the T790M mutation than afatinib. The T790M mutation rate was significantly lower in afatinib than in gefitinib and erlotinib. T790M is of great significance because osimertinib shows a good prognosis in patients with T790M mutation.

**Systematic Review Registration:**

PROSPERO, identifier CRD42021257824.

## 1 Introduction

Lung cancer is associated with significant mortality rates worldwide. Non-small-cell lung cancer (NSCLC) accounts for approximately 80% of all lung cancer cases, and its treatment depends on the stage and gene profiles of the tumors (1). Most patients with NSCLC are at an advanced stage at the time of diagnosis, have unresectable tumors, and usually present with a poor prognosis (1). Therefore, targeted therapy and chemotherapy are major treatments for these patients (1).

Traditionally, chemotherapy has been the standard treatment for patients with NSCLC. However, chemotherapy often causes serious adverse reactions and complications that can render patients unable to receive a complete course of treatment. Adenocarcinomas account for 80% of all NSCLC cases (1). Epidermal growth factor receptor (*EGFR*) mutations occur in approximately 50% of Asian and 20% of Caucasian patients with lung adenocarcinoma (2). Oral EGFR-tyrosine kinase inhibitors (EGFR-TKIs) have become promising treatments for patients with adenocarcinoma because of their good curative effects and few adverse reactions.

EGFR is a tyrosine kinase receptor that plays a key role in tumor cell proliferation and vascularization. Hence, it is an important molecular target in cancer treatment. Previous studies have shown that EGFR-TKIs are superior to paclitaxel/carboplatin in NSCLC patients with *EGFR*-sensitizing gene mutations. This finding implies that the effective treatment of NSCLC consists of EGFR-TKIs. Currently, the available EGFR-TKIs for NSCLC are first- (gefitinib, erlotinib, and icotinib), second- (afatinib and dacomitinib), and third-generation TKIs (osimertinib) (3).

Gefitinib was approved for patients with advanced NSCLC and sensitive *EGFR* mutations in July 2015 (4). Gefitinib as the first line of treatment for NSCLC patients with sensitive *EGFR* mutations showed an objective response rate (ORR) of 62–71%, progression-free survival (PFS) of 8–13 months, and overall survival (OS) of 21–30 months (4). Erlotinib was approved in 2004 for patients harboring EGFR exon 21 L858R mutations and exon 19 deletions (5). Erlotinib as the first line of treatment for NSCLC patients with sensitive *EGFR* gene mutations revealed an ORR of 58–83%, PFS of 9.7–13 months, and OS of 23–33 months (5). Afatinib is an irreversible covalent inhibitor of the ErbB receptor family, which includes EGFR, ErbB2/HER2, and ErbB4/HER4 (6). It was approved by the FDA for treating NSCLC patients with exon 21 L858R substitutions and exon 19 deletions in 2013 and for uncommon *EGFR* mutations such as L861Q in exon 21 and G719X in exon 18 in 2018 (6). Afatinib, as the first line of treatment for NSCLC patients with sensitive *EGFR* gene mutations, showed an ORR of 70%–81.8%, PFS of 13.4–15.2 months, and OS of 27.9–49 months (6).

Predictive biomarkers are important for the treatment of NSCLC. In previous studies, PDL-1 expression was found to be a predictive biomarker for the therapeutic response to immunotherapy (7). The clinical outcomes of patients with higher PDL-1 expression were better PFS and OS associated with immunotherapy (7). However, evidence shows that patients with metastatic squamous cell lung cancer tend to benefit from immunotherapy, regardless of PD-L1 status (7). Tumor mutational burden (TMB) also serves as a predictive biomarker for immunotherapy, and OS was in favor of chemotherapy for patients with low TMB and immunotherapy for patients with high TMB (7). Previous evidence suggests that micro RNAs may serve as biomarkers of response to cancer treatment and enable better management decisions (8). Furthermore, micro RNAs can be used as biomarkers for lung cancer screening and are associated with OS (8). *EGFR* mutations are the most important biomarkers for predicting treatment response to EGRF-TKIs. *EGFR* gene mutations mainly occur in the 18–21 exon and classical mutations refer to deletions in exon 19 and point mutation L858R in exon 21, which account for approximately 85% of all *EGFR* mutations (9). These mutations are associated with sensitivity to EGFR-TKIs such as gefitinib, erlotinib, afatinib, and icotinib (9, 10). EGFR-TKIs have better outcomes than chemotherapy as the first line of treatment in patients with *EGFR*-mutant NSCLC (10).

Although these EGFR-TKIs show good responses in NSCLC patients with *EGFR*-sensitizing genes (3), all treated patients eventually develop acquired resistance. The mechanisms of acquired resistance to first- or second-generation EGFR-TKIs include the T790M mutation, ERBB2 amplification, MET amplification, and transformation to small-cell lung cancer, of which T790M mutations are the most common resistance mechanism (11). The main process of developing T790M is a single nucleotide transition mutation in *EGFR*, a cytosine to thymine (C>T) mutation at position 2369, causing a threonine to methionine amino acid change at codon 790 (12). The T790M mutation leads to steric hindrance, increased binding affinity for ATP, and downstream signal transduction. When encountering patients with T790M, physicians can choose osimertinib as further therapy. As second-line therapy for NSCLC patients with acquired T790M mutations, osimertinib has better outcomes than platinum-based chemotherapy (13). Therefore, research on T790M mutation rate is of great significance because it may be related to further treatment strategies and prognosis. Therefore, T790M also serves as a biomarker for resistance to first- and second-generation EGFR-TKIs and sensitivity to osimertinib (14). The prognosis was better for patients with acquired T790M who were treated with osimertinib than for those treated with chemotherapy (13, 15–17). In patients without acquired T790M, PFS with chemotherapy is worse (13, 17). Therefore, T790M mutation is a prognostic factor.

It is important to understand the T790M mutation rate in patients with NSCLC after treatment with EGFR-TKIs. Many studies have been conducted on the T790M mutation rate after treatment with EGFR-TKIs. These studies suggest that the acquired T790M mutation rate is approximately 50%–60% (11), and the acquired T790M mutation rate with afatinib is lower than that with gefitinib or erlotinib (11). However, the range of positive rates for acquired T790M was considerably wide in these studies. In addition, many of these data come from studies with very small sample sizes, some even fewer than ten patients. Therefore, such statistics produce significant errors and no definite conclusions can be obtained.

Due to the very small number of subjects in these studies and the wide range of T790M mutation rates, it is difficult to determine whether the T790M mutation rate after afatinib treatment is lower than that after first-generation EGFR-TKIs. To solve this problem, we conducted a meta-analysis to analyze the T790M mutation rate after treatment with first- and second-generation EGFR-TKIs using direct and indirect comparisons.

## 2 Materials and Methods

### 2.1 Study Design and Participants

This study was performed in accordance with the Preferred Reporting Items for Systematic Reviews and Meta-Analyses (PRISMA) extension guidelines for network meta-analysis (18). A prospective protocol was created in advance and registered on the International Prospective Register of Systematic Reviews PROSPERO website (registration number: CRD42021257824).

### 2.2 Search Strategy

We performed a comprehensive literature search of electronic databases, including Embase, Cochrane Library, PubMed, and ClinicalTrials.gov, from their inception until May 31, 2021, without language restrictions. We aimed to compare the acquired T790M acquisition rates after treatment with different first-generation (gefitinib, erlotinib, and icotinib) and second-generation (afatinib and dacomitinib) EGFR-TKIs in patients with NSCLC. The detailed definitions of PICOS are listed in **Table S1**. The full details of the search strategy are listed in **Table S2**.

### 2.3 Study Selection Criteria

Studies were included under the following conditions: (1) observational studies, including prospective and retrospective cohort studies; (2) patients with NSCLC treated with only one EGFR-TKI during the study; (3) reported acquired T790M acquisition rates in separate EGFR-TKI groups; and (4) published as full-length articles. The exclusion criteria were (1) case-control studies or case reports; (2) T790M acquisition detected before EGFR-TKI treatment; (3) patients administered more than one EGFR-TKI; (4) EGFR-TKIs combined with chemotherapy or anti-vascular endothelial growth factor therapy; and (5) published articles, posters, or abstracts with limited information that could not be used for analysis. Bibliographies of the included studies and related systematic review articles were manually reviewed for relevant references. Two reviewers (PCH and YKW) independently reviewed the titles and abstracts of identified articles. Discrepancies or issues between reviewers were resolved by consulting a third reviewer (CCL) as an arbiter.

### 2.4 Data Extraction

A predetermined form was used by two reviewers (PCH and YKW) independently for data extraction of the following information: (1) publication year, (2) authors, (3) countries where the research was conducted, (4) NSCLC stages, (5) EGFR-TKIs, (6) number of patients with acquired resistance, (7) baseline characteristics and outcomes (sex, age, L858R mutation, exon 19 deletion, PFS, and OS); (8) biopsy sample types for examination; and (9) detection methods of T790M acquisition.

### 2.5 Outcome Measurement

The outcome was the acquired T790M acquisition rate in the research cohort after first- or second-generation EGFR-TKI treatment.

### 2.6 Data Synthesis and Statistical Analyses

We summarized the odds ratio (OR) with a 95% confidence interval (CI) as the effective size for measuring the acquired T790M acquisition rate. All graph generation and statistical analyses were performed using the statistical software RStudio (version 1.4.1106) (19). To compare the acquired T790M acquisition rate between the target EGFR-TKIs, network meta-analyses were conducted using “netmeta”, “ggplot2”, and “reshape2” packages. A random-effects network meta-analysis was performed using a consistency model. Single-arm meta-analyses with random-effects models were conducted using “meta” and “metafor” packages to estimate the specific acquired T790M acquisition rate of the target EGFR-TKIs. Subgroup analyses with Asian or Caucasian populations were conducted because of the varying characteristics of different races. Q and I^2^ statistics were used to quantify heterogeneity among the included studies.

### 2.7 Publication Bias, Direct Evidence Plot, Inconsistency Assessment, Meta-Regression, and Influence Analysis

If more than 10 studies were included in the analysis, a funnel plot was used to examine publication bias. We performed Egger’s test to assess the existence of bias in small-sample studies. Within the network meta-analysis results, a plot of direct evidence proportions was constructed to quantify the percentage of direct and indirect evidence proportions for each network estimate (20). Inconsistent assumptions were assessed using a node‐splitting model and design‐by‐treatment interaction model. Within the single-arm meta-analysis, a meta-regression analysis was performed to explore the potential associations between the effect size and target EGFR-TKIs. If more than two studies were included in the single-arm meta-analysis, an influence analysis was performed using the leave-one-out method.

### 2.8 Risk of Bias Assessment

Two reviewers (PCH and YKW) independently assessed the methodological quality of the retrieved multi-cohort studies using the ROBINS-I tool (21), and discrepancies were resolved by a third reviewer (CCL).

## 3 Results

### 3.1 Study Identification

**Figure 1:**
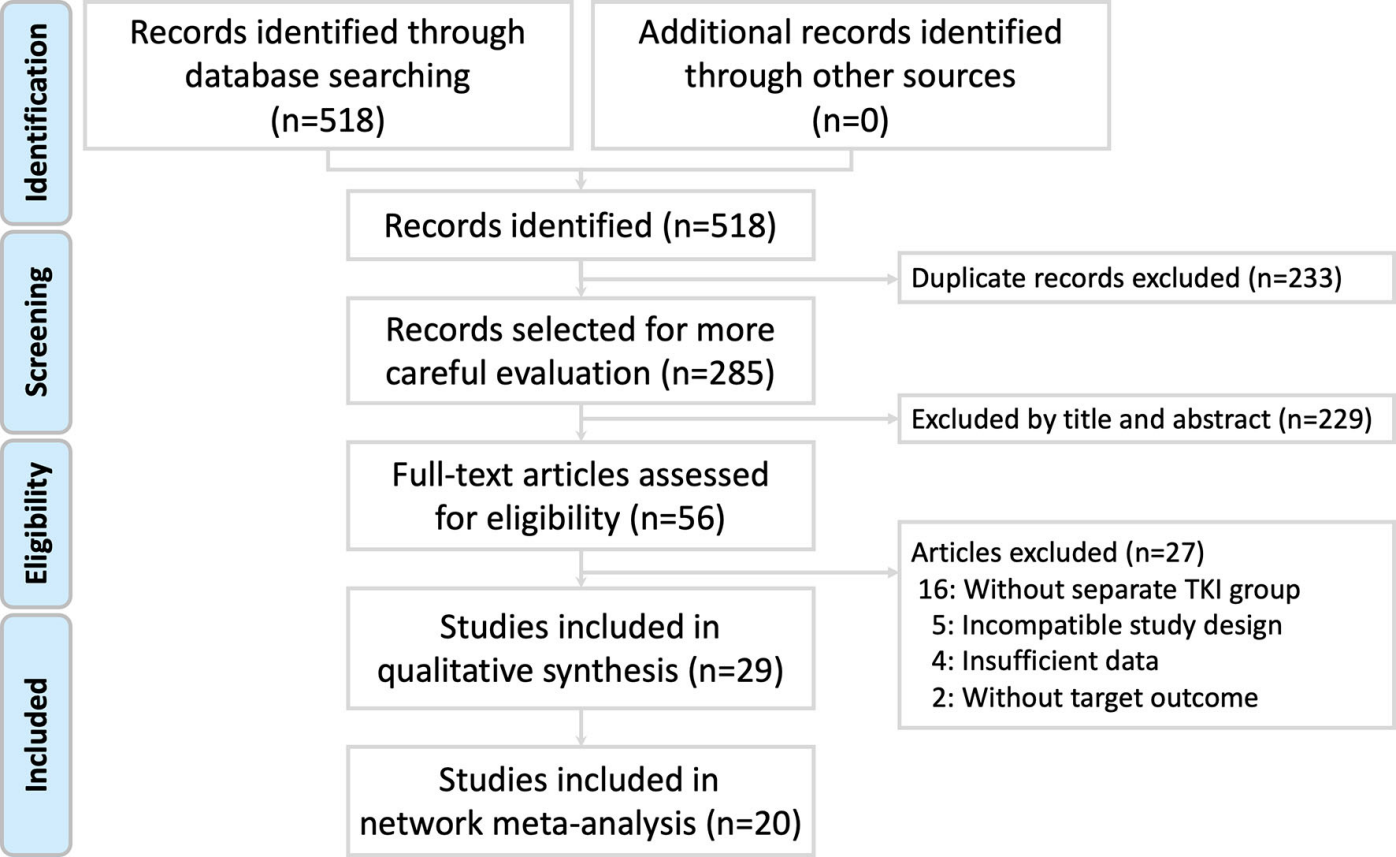
Study flow diagram.

The review process is illustrated in **Figure 1**. A total of 518 studies were identified using the search terms in the electronic databases, with 200 studies on PubMed, 265 on Embase, 31 on Cochrane Library, and 22 on ClinicalTrials.gov (**Table S2**). After removing duplicate studies and excluding titles and abstracts, 56 studies were considered for full-text evaluation, and 27 studies were excluded for different reasons (**Table S3**). Finally, 29 studies [including 23 multi-cohort studies (5, 11, 22–42) and six single-cohort studies (43–48)] were included in the risk of bias assessment and single-arm meta-analysis, and 20 multi-cohort studies were included in the network meta-analysis. Among the studies identified in the search results, acquired T790M acquisition rates after treatment with gefitinib, erlotinib, icotinib, or afatinib were noted. To our knowledge, no study has reported the acquired T790M acquisition rate after dacomitinib treatment. A summary of the retrieved studies is shown in **Table 1**.

**Table 1:** Summary of the retrieved studies.

Author, year	Study design	Country	Stage	EGFR-TKIs				Patientwith AR, n	Female,n (%)	Age, median (range),mean ± SD, y	Re-biopsysample	Detection method	Ref.
**Single-cohort study**													
Onitsuka 2010	Pro	Japan	IA-IV	G				10	7 (70.0)	61.5 (53-85)	Tissue	PCR	(43)
Uramoto 2012	Retro	Japan	IA-IV	G				19	14 (73.7)	65.0 (52-87)	Tissue	PCR	(44)
Ji 2013	Retro	Korea	N/A	G				26	16 (61.5)	58.0 (40-80)	Tissue	multiplexed PCR	(45)
Campo 2016	Pro	USA	advanced or recurrent				A	24	18 (75.0)	57 (27-83)	Tissue	PCR	(46)
Liang 2017	Retro	Taiwan	IIIB-IV				A	140	87 (62.1)	61 (28–87)	Tissue	MALDI-TOF MS	(47)
Tanaka 2017	Retro	Japan	advanced or recurrent				A	37	15 (40.5)	65 (34-79)	Tissue, Fluid	PNA-LNA PCR, Cycleave PCR, dPCR, ARMS, Cobas	(48)
**Multi-cohort study**													
Sequist 2011	Retro	USA	N/A	G	E			37	22 (59.5)	59.0 (37-88)	Tissue	multiplexed PCR	(22)
Yano 2011	Retro	Japan	N/A	G	E			22	14 (63.6)	59.5 (32-85)	Tissue	PCR	(23)
Hata 2013	Retro	Japan	N/A	G	E			78	54 (69.2)	N/A	Tissue	PNA-LNA PCR	(24)
Sun 2013	Pro	Korea	advanced or recurrent	G	E			70	52 (74.3)	N/A	Tissue	PCR	(25)
Li 2014	Pro	China	IV	G	E	I		54	25 (46.3)	51.2 (45.9-67.3)	Tissue	PCR	(26)
Jin 2016	Retro	China	IV	G	E	I		83	47 (56.6)	61 (29-85)	Tissue, Fluid	targeted pan-cancer NGS	(27)
Ko 2016	Retro	Japan	N/A	G	E		A	61	44 (72.1)	64 (39-84)	Tissue, Fluid	PCR	(28)
Matsuo 2016	Retro	Japan	advanced or recurrent	G	E		A	73	57 (78.1)	67 (48-82)	Tissue	dPCR	(29)
Nosaki 2016	Retro	Japan	advanced or metastatic	G	E		A	395	241 (61.0)	63 (27-84)	Tissue	N/A	(30)
Takahama 2016	Pro	Japan	IIIB-IV	G	E		A	260	182 (70.0)	68 (36–90)	Plasma	ddPCR	(31)
Tseng 2016	Retro	Taiwan	advanced	G	E		A	98	61 (62.2)	57.5 (30–83)	Tissue, Fluid	MALDI-TOF MS	(32)
Lee 2017	Retro	Korea	IIIA-IV	G	E			19	12 (63.2)	58 (36-72)	Tissue	NGS	(33)
Oya 2017	Retro	Japan	III-IV	G	E		A	181	110 (60.8)	65 (35-85)	Tissue	PCR	(34)
Wang 2017	Pro	China	advanced or recurrent	G	E	I		108	53 (49.1)	57 (28–79)	Tissue, Plasma	ddPCR, ARMS	(35)
Zhang 2017	Retro	China	IIIB-IV	G	E			51	32 (62.8)	58 (30-87)	Tissue	Sanger, ARMS	(36)
Kaburagi 2018	Retro	Japan	III-IV	G	E		A	233	144 (61.8)	70 (32-93)	Tissue, Plasma	allele-specific PCR, Cobas	(37)
Lee 2019	Retro	Korea	N/A	G	E		A	116	52 (44.8)	55.8	Tissue	PNA-mediated PCR clamping	(38)
Lin 2019	Retro	Taiwan	advanced or recurrent	G				134	98 (73.1)	71 (IQR: 60–80)	Tissue	RT‐PCR	(5)
					E			68	46 (67.7)	67 (IQR: 61–73)	Tissue		
							A	99	61 (61.6)	60 (IQR: 53–71)	Tissue		
Yoon 2019	Retro	Korea	IIIB-IV	G				123	58 (47.2)	60.9 ± 11.5	Tissue	PNA-mediated PCR clamping	(39)
							A	41	20 (48.8)	59.2 ± 12.3	Tissue		
Dal Maso 2020	Retro	Italy	IIIB-IV	G	E		A	235	154 (65.5)	66 (33-92)	Tissue	Pyrosequencing, PCR, MS, NGS	(41)
Del Re 2020	Retro	Italy	IIIB-IV	G	E			42	29 (69.1)	64.1 ± 8.6	Plasma	ddPCR	s(40)
							A	41	20 (48.8)	70.5 ± 11.3	Plasma		
Wagener-Ryczek 2020	Retro	Germany	N/A	G	E		A	123	70 (56.9)	68 (40-87)	Tissue	multiplexed PCR	(11)
Oya 2021	Pro	Japan	III-IV	G	E		A	62	33 (53.2)	67 (36-80)	Tissue, Plasma	ddPCR, Cobas	(42)

A, afatinib; AR, acquired resistance; ARMS, Amplification Refractory Mutation System; Cobas, Cobas^®^ EGFR Mutation Test; dPCR, digital PCR; ddPCR, droplet digital PCR; E, erlotinib; EGFR-TKI, epidermal growth factor receptor-tyrosine kinase inhibitor; G, gefitinib; I, Icotinib; MALDI-TOF MS, matrix-assisted laser desorption ionization-time of flight mass spectrometry; MS, mass spectrometry; NGS, Next Generation Sequencing; PCR, polymerase chain reaction; PNA-LNA PCR, peptide nucleic acid-locked nucleic acids PCR; PNA-mediated PCR clamping, peptide nucleic acid-mediated PCR clamping; Pro, prospective cohort; Retro, retrospective cohort; Sanger, Sanger sequencing.

### 3.2 Characteristics of the Included Participants

The characteristics of the participants are presented in **Tables 1** and **S4**. The final quantitative analysis included 3385 participants (age: 27–93-years-old), with stages I–IV and advanced, recurrent, or metastatic NSCLC. Twenty-four studies were conducted in Asia (12 in Japan, 5 in Korea, 4 in China, and 3 in Taiwan; with 2883 Asian participants), and 5 studies were conducted in Europe and North America (2 in Italy, 1 in Germany, and 2 in the USA; with 502 Caucasian participants).

### 3.3 Outcome: Acquired T790M Mutation Rate

#### 3.3.1 Risk of Acquired T790M Mutation Rate in All Participants

**Figure 2:**
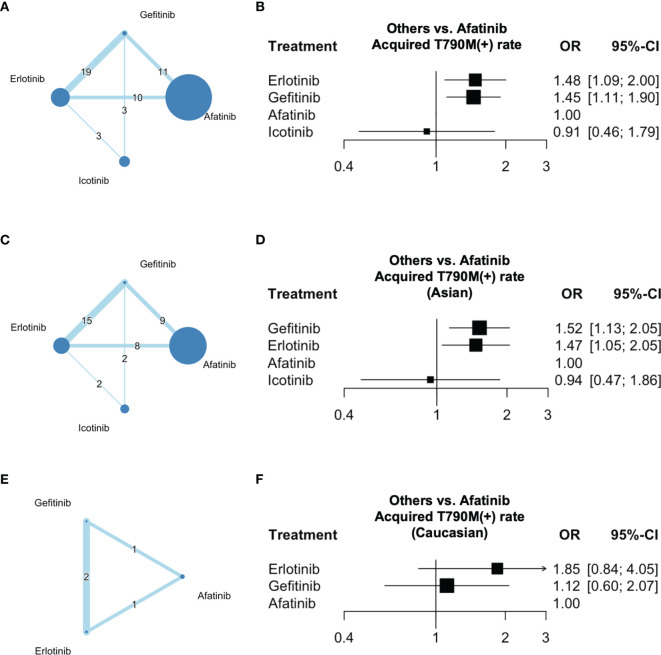
Network of the comparisons and forest plot for the network meta-analysis. Networks of eligible EGFR-TKIs comparisons for outcomes of acquired T790M mutation rate for **(A)** all participants, **(C)** Asians, and **(E)** Caucasians. Forest plots of eligible EGFR-TKI comparisons for outcomes of acquired T790M mutation rate for **(B)** all participants, **(D)** Asians, and **(F)** Caucasians. Network: The size of the nodes corresponds to the number of studies for each treatment. The lines between nodes represent a direct comparison of the trials and the thickness of the lines linking nodes corresponds to the number of trials included.

In terms of the acquired T790M acquisition rate following treatment with gefitinib, erlotinib, icotinib, and afatinib, 20 multi-cohort studies (5, 22–37, 39–41) were included in the network meta-analysis. The structure of the network is shown in **Figure 2A**. A forest plot of the network meta-analysis is shown in **Figure 2B**. There was no statistical heterogeneity among the included studies, with an I^2^ of 0% (95% CI: 0–32.6), and the Q statistic was 25.04% (*p* = 0.57) for within-design and 1.56 (*p* = 0.66) for between-designs, indicating no heterogeneity and consistency in the model used. Compared with afatinib, a higher OR of acquired T790M acquisition rate was observed after erlotinib (OR = 1.48; 95% CI: 1.09–2.00) and gefitinib (OR = 1.45; 95% CI: 1.11–1.90) treatments. The results also indicated an even OR of acquired T790M acquisition rate after treatment with icotinib (OR = 0.91, 95% CI: 0.46–1.79) compared with that after afatinib treatment in NSCLC patients. According to the league table (**Table 2**) and P-scores (**Table S5**), erlotinib was associated with the highest risk of acquired T790M acquisition rate, followed by gefitinib.

**Table 2:** League table with network meta-analysis estimates of acquired T790M mutation rate in all participants.

**Erlotinib**	1.04 (0.84 to 1.28)	1.54 (1.08 to 2.19)*	1.86 (0.78 to 4.46)
1.02 (0.83 to 1.26)	**Gefitinib**	1.44 (1.10 to 1.90)*	1.42 (0.70 to 2.91)
1.48 (1.09 to 2.00)*	1.45 (1.11 to 1.90)*	**Afatinib**	–
1.62 (0.86 to 3.05)	1.59 (0.85 to 2.98)	1.10 (0.56 to 2.15)	**Icotinib**

Pairwise (upper-right portion) and network (lower-left portion) meta-analysis results are presented as estimated effect sizes as mean difference (MD) and 95% confidence interval for the outcome of the acquired T790M mutation rate. An MD > 0 favors treatment in the column for the acquired T790M mutation rate. *Statistically significant.

#### 3.3.2 Risk of Acquired T790M Mutation Rate in Asian Patients

In terms of the acquired T790M acquisition rates in Asian patients following treatment with gefitinib, erlotinib, icotinib, and afatinib, 18 multi-cohort studies (5, 23–37, 39, 41) were included in the subgroup network meta-analysis. The structure of the network is shown in **Figure 2C**. A forest plot of the network meta-analysis is shown in **Figure 2D**. There was no statistical heterogeneity among the included studies, with I^2^ 0% (95% CI: 0–34.8), and the Q statistic was 22.35 (*p* = 0.55) for within-design and 1.70 (*p* = 0.63) for between-designs, indicating no heterogeneity and consistency in the model used. The results indicated a higher OR of acquired T790M acquisition rate after treatment with gefitinib (OR = 1.52; 95% CI: 1.13–2.05) and erlotinib (OR = 1.47; 95% CI: 1.05–2.05) than that after afatinib treatment in patients with NSCLC. Furthermore, an even OR of acquired T790M acquisition rate was observed after icotinib treatment (OR = 0.94; 95% CI: 0.47–1.86) compared with that in afatinib-treated patients with NSCLC. According to the league table (**Table 3**) and P-scores (**Table S5**), gefitinib was associated with the highest risk of acquired T790M acquisition rate, followed by erlotinib.

**Table 3:** League table with network meta-analysis estimates of acquired T790M mutation rate in Asian patients.

**Gefitinib**	1.02 (0.81 to 1.27)	1.54 (1.13 to 2.09) *	1.42 (0.70 to 2.91)
1.04 (0.83 to 1.29)	**Erlotinib**	1.47 (0.99 to 2.18)	1.86 (0.78 to 4.46)
1.52 (1.13 to 2.05)*	1.47 (1.05 to 2.05)*	**Afatinib**	–
1.62 (0.87 to 3.04)	1.57 (0.83 to 2.96)	1.07 (0.54 to 2.12)	**Icotinib**

Pairwise (upper-right portion) and network (lower-left portion) meta-analysis results are presented as estimated effect sizes as mean difference (MD) and 95% confidence interval for the outcome of the acquired T790M mutation rate. An MD > 0 favors treatment in the column for the acquired T790M mutation rate. *Statistically significant.

#### 3.3.3 Risk of Acquired T790M Mutation Rate in Caucasian Patients

In terms of the acquired T790M acquisition rate in Caucasian patients following treatment with gefitinib, erlotinib, and afatinib, two multi-cohort studies (22, 40) were included in the subgroup network meta-analysis. The structure of the network is shown in **Figure 2E**. No statistical heterogeneity was observed among the included studies, with an I^2^ value of 0%. The Q statistic was 0.11 (*p* = 0.91) for between-designs, indicating no heterogeneity and consistency in the model used. A forest plot of the network meta-analysis is shown in **Figure 2F**. The results indicated an even OR of acquired T790M acquisition rate after treatment with erlotinib (OR = 1.85; 95% CI: 0.84–4.05) and gefitinib (OR = 1.12, 95% CI: 0.60–2.07) compared with that in afatinib-treated patients with NSCLC. According to the league table (**Table 4**) and P-scores (**Table S5**), gefitinib, erlotinib, and afatinib were associated with an even risk of an acquired T790M acquisition rate.

**Table 4:** League table with network meta-analysis estimates of acquired T790M mutation rate in Caucasian patients.

**Erlotinib**	1.65 (0.85 to 3.23)	1.87 (0.83 to 4.19)
1.65 (0.85 to 3.23)	**Gefitinib**	1.11 (0.60 to 2.07)
1.85 (0.84 to 4.05)	1.12 (0.60 to 2.07)	**Afatinib**

Pairwise (upper-right portion) and network (lower-left portion) meta-analysis results are presented as estimated effect sizes as mean difference (MD) and 95% confidence interval for the outcome of the acquired T790M mutation rate. An MD > 0 favors treatment in the column for the acquired T790M mutation rate.

#### 3.3.4 Acquired T790M Mutation Rate in All Participants

**Figure 3:**
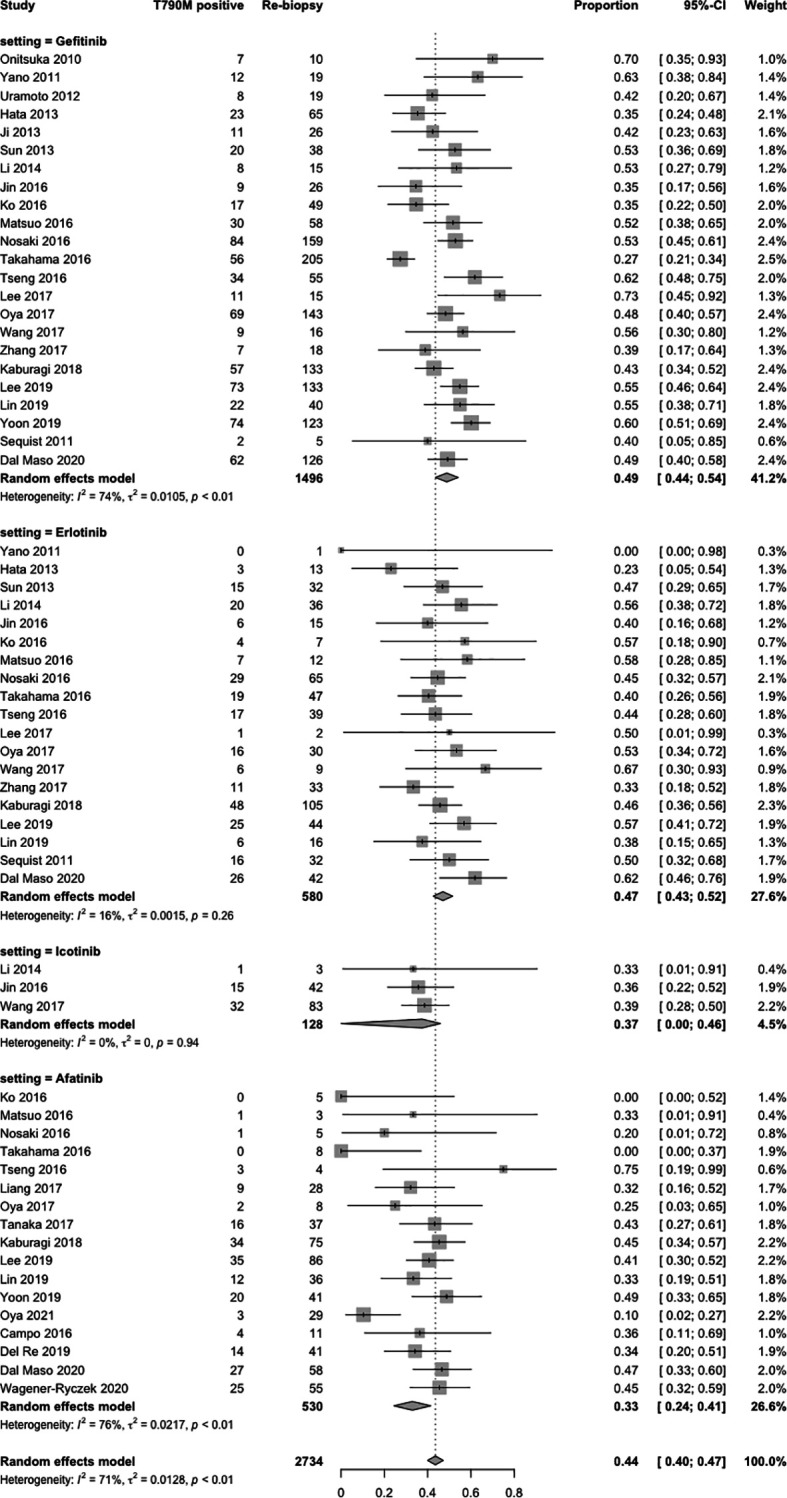
Forest plot of subgroup single-arm meta-analysis of all participants.

In terms of specific acquired T790M acquisition rates with EGFR-TKIs following treatment with gefitinib, erlotinib, icotinib, and afatinib, 29 studies (5, 11, 22–48) were included in the single-arm meta-analysis. A forest plot of the analysis is shown in **Figure 3**. The overall rate of acquired T790M acquisition was 44% (95% CI: 40–47; I^2^ = 71%). The specific acquired T790M acquisition rates were 49% for gefitinib (95% CI: 44–54; I^2^ = 74%), 47% for erlotinib (95% CI: 43–52; I^2^ = 16%), 37% for icotinib (95% CI: 0–46; I^2^ = 0%), and 33% for afatinib (95% CI, 24–41; I^2^ = 76%). The meta-regression results indicated potential associations between the acquired T790M acquisition rate and different EGFR-TKIs, with statistical significance (*p* < 0.0001) (**Table S6**).

#### 3.3.5 Acquired T790M Mutation Rate in Asian Patients

**Figure 4:**
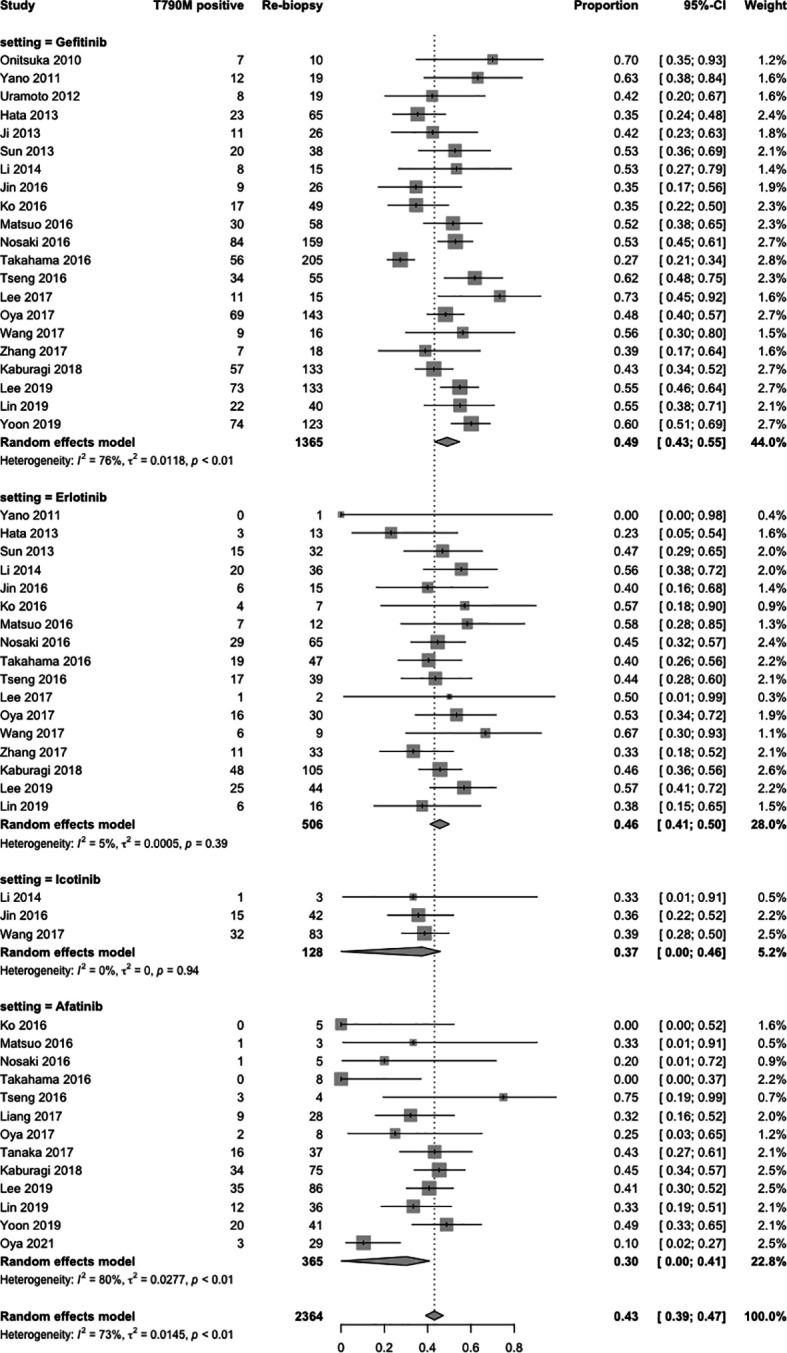
Forest plot of subgroup single-arm meta-analysis of Asian patients.

In terms of specific acquired T790M acquisition rates in Asian patients following treatment with gefitinib, erlotinib, icotinib, and afatinib, 24 studies (5, 23–39, 42–45, 47, 48) were included in the single-arm meta-analysis. A forest plot of the analysis is shown in **Figure 4**. The overall acquired T790M acquisition rate was 43% (95% CI: 39–47; I^2^ = 73%). The specific acquired T790M acquisition rates were 49% for gefitinib (95% CI: 43–52; I^2^ = 76%), 46% for erlotinib (95% CI: 41–50; I^2^ = 5%), 37% for icotinib (95% CI: 0–46; I^2^ = 0%), and 30% for afatinib (95% CI: 0–41; I^2^ = 80%). Meta-regression results indicated potential associations between the acquired T790M acquisition rate and different EGFR-TKIs in Asians, with statistical significance (*p* < 0.0001) (**Table S6**).

#### 3.3.6 Acquired T790M Mutation Rate in Caucasian Patients

**Figure 5:**
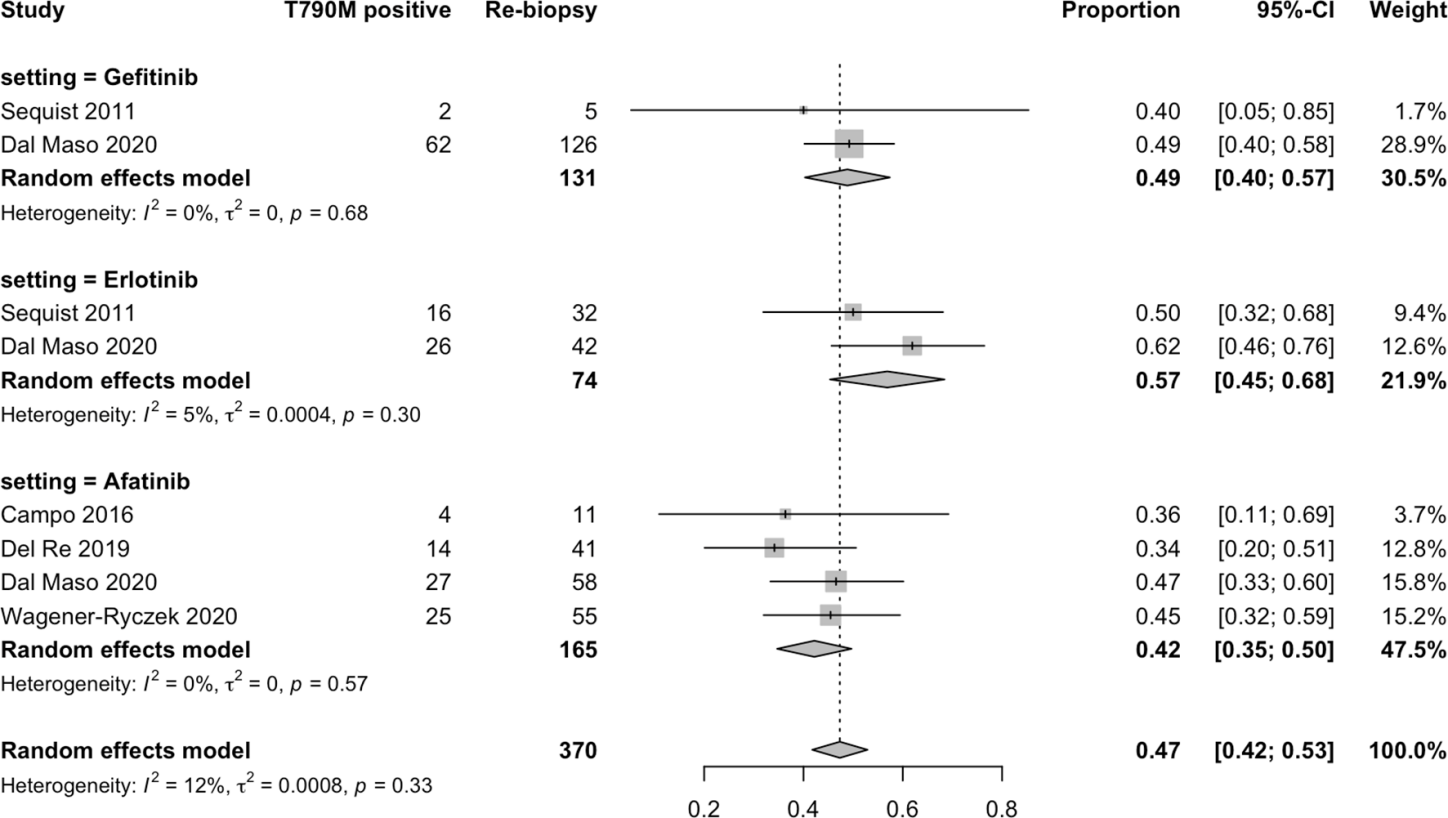
Forest plot of subgroup single-arm meta-analysis of Caucasian patients.

In terms of the acquired T790M acquisition rate of EGFR-TKIs in Caucasian patients following treatment with gefitinib, erlotinib, and afatinib, five studies (11, 22, 40, 41, 46) were included in the single-arm meta-analysis. A forest plot of the analysis is shown in **Figure 5**. The overall acquired T790M acquisition rate was 47% (95% CI: 42–53; I^2^ = 12%). The specific acquired T790M acquisition rates were 49% for gefitinib (95% CI: 40–57; I^2^ = 0%), 57% for erlotinib (95% CI: 45–68; I^2^ = 0%), and 42% for afatinib (95% CI: 35–50; I^2^ = 0%). Meta-regression results indicated no potential association between the acquired T790M acquisition rate and different EGFR-TKIs in Caucasians (*p* = 0.6621) (**Table S6**).

#### 3.3.7 Meta-Regression Analysis of Gefitinib, Erlotinib, and Afatinib in Asians/Caucasians

We also conducted a meta-regression analysis to investigate the potential associations between gefitinib, erlotinib, and afatinib treatments and Asians/Caucasians. In terms of the acquired T790M acquisition rate of gefitinib, the results indicated potential associations between the acquired T790M acquisition rate and Asian/Caucasian populations (*p* < 0.0001). In terms of the acquired T790M acquisition rate of erlotinib, the results indicated no potential association between the acquired T790M acquisition rate and Asians/Caucasians (*p* = 0.3941). In terms of the acquired T790M acquisition rate of afatinib, the results indicated potential associations between the acquired T790M acquisition rate and Asian/Caucasian populations (*p* < 0.0001). Gefitinib and afatinib treatments had different effects on the acquired T790M acquisition rates in Asians and Caucasians (**Table S7**).

#### 3.3.8 Detection of Acquired T790M Mutation Between Tissue or Plasma Biopsy

We conducted single-arm meta-analyses and meta-regression analyses to evaluate the rate of acquired T790M acquisition in tissue or plasma biopsy samples. Forest plots of the analyses are shown in **Figure S1**. The results showed that after treatment with gefitinib, the acquired T790M acquisition rate was 52% (95% CI: 48–56; I^2^ = 30%) in tissue biopsy samples, whereas the acquired T790M acquisition rate was 27% (95% CI: 21–33) in plasma biopsy samples. After treatment with erlotinib, the acquired T790M acquisition rate was 46% (95% CI: 40–52; I^2^ = 22%) in tissue biopsy samples, whereas the acquired T790M acquisition rate was 40% (95% CI: 26–54) in plasma biopsy samples. Furthermore, after treatment with afatinib, the acquired T790M acquisition rate was 39% (95% CI: 34–45; I^2^ = 0%) in tissue biopsy samples, whereas the acquired T790M mutation rate was 33% (95% CI: 23–42; I^2^ = 65%) in plasma biopsy samples. Meta-regression analysis results showed potential associations between the acquired T790M mutation rate and tissue/plasma biopsy after treatment with gefitinib, erlotinib, or afatinib (**Table S8**). After treatment with gefitinib, erlotinib, or afatinib, the detection rate of the acquired T790M mutation was significantly lower in plasma biopsy samples than that in tissue biopsy samples.

### 3.4 Publication Bias

Among the network meta-analyses of all Asian patients, funnel plots of publication bias showed general symmetry. Egger’s test showed no significant publication bias among the included studies (**Figures S2A, B**). Because only two studies were included in the network meta-analysis for Caucasians, no further assessment of publication bias was performed.

In the single-arm meta-analysis of all participants, funnel plots of publication bias showed general symmetry (**Figure S3A**). Because the intercept was close to zero, the small study bias was not significant (**Figure S3B**). In the single-arm meta-analysis for Asians, funnel plots of publication bias showed a general symmetry (**Figure S3C**). Because the intercept was significantly close to zero, the small study bias was not significant (**Figure S3D**). As only three studies were included in the single-arm meta-analysis for Caucasians, no further assessment of publication bias was performed.

### 3.5 Direct Evidence Plots and Inconsistency Assessment

In the network meta-analyses, direct evidence plots for all patients, Asian and Caucasian are presented in **Figure S4**. We found no evidence of inconsistencies using either node-splitting (**Figure S5**) or design-by-treatment interaction model approaches (**Figure S6**).

### 3.6 Influence Analysis

In the single-arm meta-analysis for gefitinib, erlotinib, icotinib, and afatinib treatment in Asian patients, the results indicated no significant changes in the integrated or after eliminating each study individually (**Figure S7**). For the single-arm meta-analysis of afatinib in Caucasians, the results indicated no significant changes in the integrated OR while eliminating each study individually (**Figure S8**).

### 3.7 Risk of Bias Assessment: ROBINS-I

The ROBINS-I results are presented in **Table S9**. Most of the studies had a moderate risk of overall bias. There were three main reasons. (1) For baseline *EGFR* mutation types, the number of patients with L858R mutations or exon 19 deletions was not balanced or adjusted for different EGFR-TKI-treated groups. Hence, we propose a moderate risk of bias owing to confounding factors. (2) For the detection of T790M mutation, five studies used multiple methods (**Table 1**) (35–37, 41, 42). As the detection accuracy differed according to the detection method (49, 50), we proposed a moderate risk of bias in the measurement of outcomes. (3) For the re-biopsy samples, six studies used multiple types of samples, including tissue, plasma, and fluid (**Table 1**) (27, 28, 32, 35, 37, 42). As the detection accuracy differed according to the type of examination sample (50), we proposed a moderate risk of bias in the measurement of outcomes.

## 4 Discussion

This is the first meta-analysis of the acquired T790M mutation rate associated with treatment using different EGFR-TKIs, and it has revealed that the acquired T790M mutation rate was significantly lower with afatinib (33%) than that with gefitinib (49%) and erlotinib (47%) (*p* < 0.05) treatments in the overall population. The first- and second-generation EGFR-TKIs are different. The mechanisms underlying the lower T790M mutation rate after afatinib treatment are unclear; several hypotheses can explain this result.

Initially, gefitinib, erlotinib, and afatinib were considered similar EGFR-TKIs. However, gefitinib and erlotinib are reversible EGRF-TKIs with similar activities in *in vitro* and xenograft assays. In our analysis, the incidence of acquired T790M mutations was similar following treatment with gefitinib and erlotinib. In contrast, afatinib is an irreversible EGFR-TKI that inhibits the ErbB receptor family and causes rare *EGFR* mutations, including exon 18 p.G719X and exon 21 p.L861Q point mutations (6). Afatinib has pharmacological characteristics that differ from those of gefitinib and erlotinib.

El Kadi et al. found that acquired *EGFR* T790M occurs mainly through activation-induced cytidine deaminase (AICDA)-mediated deamination of 5-methylcytosine following TKI treatment (12). They reported that EGFR-TKI treatment leads to activation of the nuclear factor-kappa B pathway, which in turn induces the expression of AICDA, further causing deamination of 5-methylcytosine to thymine at position c.2369 to generate the T790M mutation. The different pharmacological characteristics of these EGFR-TKIs may lead to different rates of acquired T790M mutation. Gefitinib and erlotinib showed higher AICDA expression than afatinib (12). Therefore, it is rational to understand the higher frequencies of the T790M mutation rates following treatment with gefitinib and erlotinib.

Another hypothesis is that clonal selection during different EGFR-TKI treatments may lead to different clonality of the acquired resistance. Previous studies have shown that afatinib suppresses the growth of lung cancer cells harboring T790M cells (51, 52). Furthermore, afatinib exerts a 100-fold potent activity against T790M cell lines than first-generation EGFR-TKIs (51). Afatinib was initially considered a potential salvage therapy after first-generation TKIs. However, the clinical use of afatinib as salvage therapy after first-generation TKIs has been disappointing (52) because of the difficulty in increasing the clinical dose of afatinib to reach the afatinib concentration in the human body in an *in vitro* study (52). Although afatinib cannot effectively overcome T790M at a clinically achievable dose, it may reduce the occurrence of T790M colonies. Therefore, T790M subclonies are likely to be enriched under the different effects of gefitinib, erlotinib, and afatinib (39). Previous studies have shown that prolonged exposure to EGFR-TKIs promotes selective survival of T790M-positive cells (5, 53, 54). These results support the hypothesis of clonal selection for EGFR-TKI therapy.

Interestingly, we found that the rate of acquired T790M mutation was slightly lower in Asians (43%) than that in Caucasians (47%). Asian and Caucasian patients with lung cancer have different genetic susceptibilities (2). For example, common *EGFR* mutations in adenocarcinoma occur in approximately 50% of Asian patients and 20% of Caucasian patients (2). In contrast, the incidence of *KRAS* mutations in European populations (30%) is higher than that in Asian populations (<10%) (2). The exact mechanisms for the different *EGFR* or *KRAS* mutations in Asian and Caucasian populations remain unclear. In this meta-analysis, we found that the rate of acquired T790M mutation was higher in Caucasian patients than that in Asian patients. However, the reason for this difference remains unclear.

The clinical outcomes of EGFR-TKIs are controversial. It has been shown that for NSCLC patients with sensitive *EGFR* mutations, first-line treatment with gefitinib yielded PFS of 8–13 months (55), erlotinib yielded a PFS of 9.7–13 months (5), and afatinib yielded a PFS of 13.4–15.2 months (6). The LUX-Lung 7 trial suggested that afatinib achieved superior clinical outcomes compared with gefitinib-treated patients bearing *EGFR* L858R or exon 19 deletions (56). However, other studies have shown that PFS and OS are similar in gefitinib- and afatinib-treated patients (39, 57). In a real-world study, the clinical outcomes of PFS or OS were similar among patients treated with gefitinib, erlotinib, or afatinib for NSCLC patients bearing sensitizing *EGFR* mutations (5). Therefore, the clinical outcomes were similar among the three EGFR-TKIs. However, these EGFR-TKIs have different incidences of T790M mutation.

**Figure 6:**
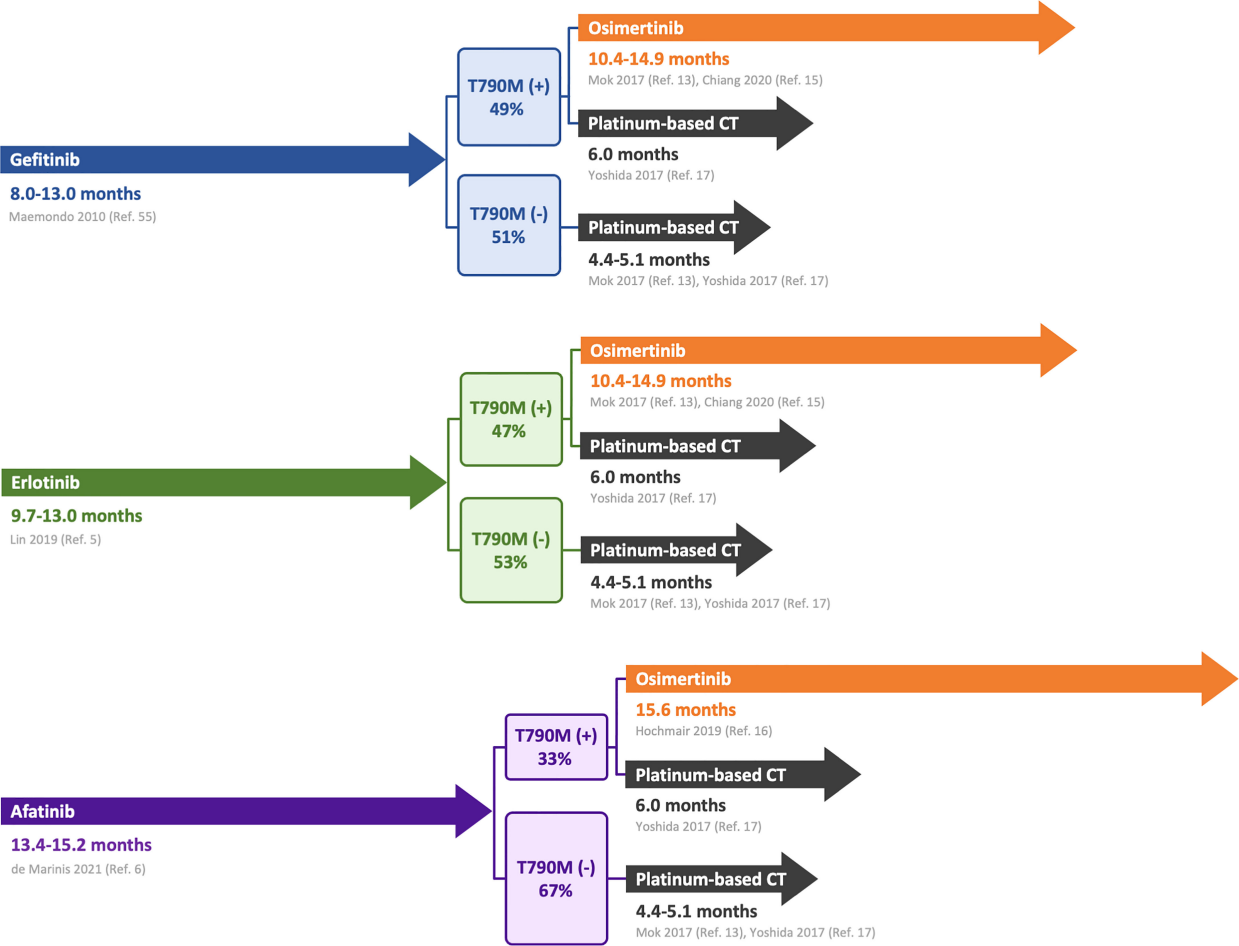
Progression-free survival of T790M-positive and -negative patients.

Patients with acquired T790M can choose osimertinib treatment, whereas those without acquired T790M can only receive chemotherapy. As shown in **Figure 6**, the clinical prognosis was better for patients with acquired T790M mutations who were treated with osimertinib. PFS of NSCLC patients with acquired T790M after treatment with gefitinib, erlotinib, and afatinib was 10.4-15.6 months (13, 15, 16). The PFS of NSCLC patients with acquired T790M mutation treated with platinum-based chemotherapy was only 6 months (17). In patients without acquired T790M, PFS after platinum-based chemotherapy was worse, only 4.4–5.1 months (13, 17). Joo et al. also suggested that osimertinib treatment was independently associated with better outcomes, such as longer OS and PFS (54). A preclinical model also suggested that T790M-positive cells grow more slowly than T790M-negative cells (53). Thus, T790M appears to be a prognostic marker. As the presence of T790M is an important factor in choosing treatment and determining prognosis, assessing which population will develop T790M is vital. This should be considered when selecting EGFR-TKIs, and patients must be screened for acquired *EGFR* T790M mutations at the time of tumor progression.

The occurrence of T790M mutation has important implications for further treatment and prognosis after first- or second-generation EGFR-TKI therapy. The PFS after afatinib treatment was similar to or slightly higher than that after gefitinib and erlotinib treatments. However, the T790M mutation rate of afatinib was significantly lower than those of gefitinib and erlotinib. Patients with acquired T790M mutations during EGFR-TKI treatment showed better PFS and OS with osimertinib treatment. Accordingly, we suggest gefitinib and erlotinib as the first-line treatments for patients with advanced NSCLC. However, in precision medicine, selecting patients with a high probability of receiving first-generation EGFR-TKIs is a better strategy. Ouyang et al. showed that a lower body mass index (≤ 25 kg/m^2^), higher levels of neuron-specific enolase (> 17.9 ng/ml), and retroperitoneal lymph node metastasis before treatment are independent risk factors for the acquired T790M mutation (58). Lin et al. revealed that the independent factors for T790M mutation were first-generation EGFR-TKIs, initial liver metastasis, male sex, and uncommon *EGFR* mutations (5).

### 4.1 Limitations of This Study

This meta-analysis had several limitations. First, the literature cited in this meta-analysis was retrospective but not a randomized control trial. The clinical conditions of the subjects who received the three EGFR-TKIs were unequal. Second, the study was not designed to determine the incidence of T790M mutations. Therefore, the timing of biopsy, sample collection (tissues or blood), and methods for detecting T790M were not well designed. Our analysis showed a significantly lower detection rate of acquired T790M mutations in plasma biopsy samples than that in tissue biopsy samples. There are many methods for detecting the T790M mutation. Droplet digital PCR (ddPCR) and matrix-assisted laser desorption/ionization-time of flight mass spectrometry (MALDI-TOF MS) are highly sensitive approaches capable of detecting mutations, and studies using ddPCR and MALDI-TOF MS have shown a higher incidence of detecting T790M (40, 47). The duration of exposure to EGFR-TKIs is an independent factor for the occurrence of T790M mutations. However, the time required for rebiopsy has not been standardized. Third, we included all the available first- and second-generation EGFR-TKIs. However, studies on acquired T790M in patients treated with icotinib or dacomitinib are limited. Therefore, we did not discuss the effects of icotinib or dacomitinib. Further meta-analysis should be performed with more studies on icotinib and dacomitinib.

## 5 Conclusions

Lung cancer is associated with significant mortality rates worldwide. Lung adenocarcinoma is the most common type of NSCLC. *EGFR* mutations occur frequently in adenocarcinoma, and oral EGFR-TKIs with good responses are promising treatments for patients with advanced NSCLC. T790M mutation is the most common mechanism of acquired resistance. Our meta-analysis of 29 studies showed that erlotinib and gefitinib had a higher OR for the T790M mutation than afatinib. The acquired T790M mutation rate was significantly lower with afatinib treatment than that with gefitinib or erlotinib in the overall population. The T790M mutation rate is of great significance because osimertinib treatment in patients with the T790M mutation shows a good prognosis.

## Data Availability Statement

The original contributions of this study are included in the article/**Supplementary Material**. Further inquiries can be directed to the corresponding author.

## Author Contributions

The authors confirm their contribution to the paper as follows: study conception and design: Y-KW and C-CL. Data collection: P-CH and Y-KW. Statistical analysis: P-CH, C-YK, and I-ST. Interpretation of results: Y-KW, C-YH, M-CY, and C-CL. Drafting manuscript: P-CH and C-CL. And project administration: C-CL. All authors have reviewed the results and approved the final version of the manuscript.

## Funding

This study was supported by grants from Taipei Tzu Chi Hospital and Buddhist Tzu Chi Medical Foundation [TCRD-TPE-109-24(3/3) and TCRD-TPE-111-11].

## Conflict of Interest

The authors declare that the research was conducted in the absence of any commercial or financial relationships that could be construed as potential conflicts of interest.

## Publisher’s Note

All claims expressed in this article are solely those of the authors and do not necessarily represent those of their affiliated organizations, or those of the publisher, the editors and the reviewers. Any product that may be evaluated in this article, or claim that may be made by its manufacturer, is not guaranteed or endorsed by the publisher.
