# Supplementary material for: Comparison of T790M Acquisition After Treatment With First- and Second-Generation Tyrosine-Kinase Inhibitors: A Systematic Review and Network Meta-Analysis
Source: Front Oncol. 2022 Jun 28;12:869390. doi: 10.3389/fonc.2022.869390 (PMC9274284; doi:10.3389/fonc.2022.869390)

Supplementary Materials

Comparison of T790M Acquisition After First- and Second-generation Tyrosine-Kinase Inhibitors: A Systematic Review and Network Meta-analysis

Po-Chun Hsieh, Yao-Kuang Wu, Chun-Yao Huang, Mei-Chen Yang, Chan-Yen Kuo, I-Shiang Tzeng, Chou-Chin Lan*

Supplementary Tables	Page
Table S1. Definition of the PICOS in the current study	2
Table S2. Search strategy and results	3
Table S3. List of excluded articles with reasons	5
Table S4. Patient characteristics in the retrieved studies	8
Table S5. P-score of the EGFR-TKIs in the network meta-analyses	9
Table S6. Meta-regression model with univariate analysis of EGFR-TKIs	10
Table S7. Meta-regression model with univariate analysis of Asian and Caucasian	11
Table S8. Meta-regression model with univariate analysis of tissue and plasma biopsy	12
Table S9. ROBINS-I risk of bias assessment for multi-cohort studies of acquired T790M mutation rate	13

Supplementary Figures	Page
Figure S1. Acquired T790M mutation rate detected by tissue or plasma biopsy after treatment with (A) Gefitinib; (B) Erlotinib; (C) Afatinib.	14
Figure S2. Funnel plots of the network meta-analysis of studies for (A) all participants ;(B) Asians.	15
Figure S3. Funnel plots of the single-arm meta-analysis of studies for (A) all participants ;(C) Asians. Small study bias of the single-arm meta-analysis of studies for (B) all participants ;(D) Asians.	16
Figure S4. Direct evidence plots of the network meta-analysis for (A) all participants ;(B) Asians ;(C) Caucasian.	17
Figure S5. Node-splitting analysis of the network meta-analysis for (A) all participants ;(B) Asians ;(C) Caucasian.	18
Figure S6. Design-by-treatment interaction model of the network meta-analysis for (A) all participants ;(B) Asians ;(C) Caucasian.	19
Figure S7. Influence analysis of the single-arm meta-analysis of Asians for (A) gefitinib ;(B) erlotinib ;(C) icotinib; (D) afatinib.	20
Figure S8. Influence analysis of the single-arm meta-analysis of Caucasians for afatinib.	21

Table S1. Definition of the PICOS in the current study

Patient	EGFR-mutant non-small cell lung cancer patients
Intervention/ Comparison	Administered with only one EGFR TKI (first-generation [gefitinib, erlotinib, icotinib] or second-generation [afatinib, dacomitinib]) during the study.
Outcome	Acquired T790M mutation rate in separate EGFR-TKI groups
Study design	Observational study (including prospective and retrospective cohort study)

Table S2. Search strategy and results

Search strategy and results in PubMed		
Search	Query	Results
#1	"carcinoma, non small cell lung"[MeSH Terms]	57,156
#2	"gefitinib"[MeSH Terms] OR "gefitinib"[All Fields] OR "gefitinib s"[All Fields] OR ("erlotinib hydrochloride"[MeSH Terms] OR ("erlotinib"[All Fields] AND "hydrochloride"[All Fields]) OR "erlotinib hydrochloride"[All Fields] OR "erlotinib"[All Fields] OR "erlotinib s"[All Fields]) OR ("icotinib"[Supplementary Concept] OR "icotinib"[All Fields]) OR ("afatinib"[MeSH Terms] OR "afatinib"[All Fields]) OR ("dacomitinib"[Supplementary Concept] OR "dacomitinib"[All Fields])	13,382
#3	"T790M"[All Fields]	2,318
#4	#1 AND #2 AND #3	480
#5	#4 AND acquired resistance	244
#6	#5 NOT osimertinib	200
**Search strategy and results in Embase**		
Search	Query	Results
#1	‘Carcinoma, Non Small Cell Lung’ OR ‘Carcinomas, Non-Small-Cell Lung’ OR ‘Lung Carcinoma, Non-Small-Cell’ OR ‘Lung Carcinomas, Non-Small-Cell’ OR ‘Non-Small-Cell Lung Carcinomas’ OR ‘Non-Small-Cell Lung Carcinoma’ OR ‘Non Small Cell Lung Carcinoma’ OR ‘Carcinoma, Non-Small Cell Lung’ OR ‘Non-Small Cell Lung Carcinoma’ OR ‘Non-Small Cell Lung Cancer’ OR ‘Nonsmall Cell Lung Cancer’	179,427
#2	‘Gefitinib’ OR ‘Erlotinib’ OR ‘ Icotinib’ OR ‘Afatinib’ OR ‘Dacomitinib’	44,526
#3	‘T790M’	5,184
#4	#1 AND #2 AND #3	2,262
#5	#4 AND acquired resistance	1,004
#6	#5 NOT osimertinib	635
#7	#6 AND 'human'/de AND 'article'/it	265
**Search strategy and results in Cochrane Library**		
Search	Query	Results
#1	‘Carcinoma, Non Small Cell Lung’ OR ‘Carcinomas, Non-Small-Cell Lung’ OR ‘Lung Carcinoma, Non-Small-Cell’ OR ‘Lung Carcinomas, Non-Small-Cell’ OR ‘Non-Small-Cell Lung Carcinomas’ OR ‘Non-Small-Cell Lung Carcinoma’ OR ‘Non Small Cell Lung Carcinoma’ OR ‘Carcinoma, Non-Small Cell Lung’ OR ‘Non-Small Cell Lung Carcinoma’ OR ‘Non-Small Cell Lung Cancer’ OR ‘Nonsmall Cell Lung Cancer’	13,686
#2	‘Gefitinib’ OR ‘Erlotinib’ OR ‘ Icotinib’ OR ‘Afatinib’ OR ‘Dacomitinib’	2,740
#3	‘T790M’	294
#4	#1 AND #2 AND #3	155

#5	#4 AND acquired resistance	46
#6	#5 NOT osimertinib	31
**Search strategy and results in ClinicalTrials.gov**		
Search	Query	Results
#1	[Condition or disease]<br>‘Carcinoma, Non Small Cell Lung’ OR ‘Carcinomas, Non-Small-Cell Lung’ OR ‘Lung Carcinoma, Non-Small-Cell’ OR ‘Lung Carcinomas, Non-Small-Cell’ OR ‘Non-Small-Cell Lung Carcinomas’ OR ‘Non-Small-Cell Lung Carcinoma’ OR ‘Non Small Cell Lung Carcinoma’ OR ‘Carcinoma, Non-Small Cell Lung’ OR ‘Non-Small Cell Lung Carcinoma’ OR ‘Non-Small Cell Lung Cancer’ OR ‘Nonsmall Cell Lung Cancer’	5,530
#2	[Intervention/treatment]<br>‘Gefitinib’ OR ‘Erlotinib’ OR ‘ Icotinib’ OR ‘Afatinib’ OR ‘Dacomitinib’	1,392
#3	[Other terms]<br>‘T790M’	175
#4	#1 AND #2 AND #3	22

Table S3. List of excluded articles with reasons

Without separate TKI group (n=16)	
#	Title
1	Nakamura T, Sueoka-Aragane N, Iwanaga K, et al. A noninvasive system for monitoring resistance to epidermal growth factor receptor tyrosine kinase inhibitors with plasma DNA. *J Thorac Oncol*. 2011;6(10):1639-1648. doi:10.1097/JTO.0b013e31822956e8
2	Oxnard GR, Arcila ME, Sima CS, et al. Acquired resistance to EGFR tyrosine kinase inhibitors in EGFR-mutant lung cancer: distinct natural history of patients with tumors harboring the T790M mutation. *Clin Cancer Res*. 2011;17(6):1616-1622. doi:10.1158/1078-0432.CCR-10-2692
3	Kuiper JL, Heideman DA, Thunnissen E, et al. Incidence of T790M mutation in (sequential) rebiopsies in EGFR-mutated NSCLC-patients. *Lung Cancer*. 2014;85(1):19-24. doi:10.1016/j.lungcan.2014.03.016
4	Chen Q, Quan Q, Ding L, et al. Continuation of epidermal growth factor receptor tyrosine kinase inhibitor treatment prolongs disease control in non-small-cell lung cancers with acquired resistance to EGFR tyrosine kinase inhibitors. *Oncotarget*. 2015;6(28):24904-24911. doi:10.18632/oncotarget.4570
5	Otsuka K, Hata A, Takeshita J, et al. EGFR-TKI rechallenge with bevacizumab in EGFR-mutant non-small cell lung cancer. *Cancer Chemother Pharmacol*. 2015;76(4):835-841. doi:10.1007/s00280-015-2867-8
6	Del Re M, Tiseo M, Bordi P, et al. Contribution of KRAS mutations and c.2369C > T (p.T790M) EGFR to acquired resistance to EGFR-TKIs in EGFR mutant NSCLC: a study on circulating tumor DNA. *Oncotarget*. 2017;8(8):13611-13619. doi:10.18632/oncotarget.6957
7	Sueoka-Aragane N, Katakami N, Satouchi M, et al. Monitoring EGFR T790M with plasma DNA from lung cancer patients in a prospective observational study. *Cancer Sci*. 2016;107(2):162-167. doi:10.1111/cas.12847
8	Yoshida T, Kuroda H, Oya Y, et al. Clinical outcomes of platinum-based chemotherapy according to T790M mutation status in EGFR-positive non-small cell lung cancer patients after initial EGFR-TKI failure. *Lung Cancer*. 2017;109:89-91. doi:10.1016/j.lungcan.2017.05.001
9	Nakamura T, Nakashima C, Komiya K, et al. Mechanisms of acquired resistance to afatinib clarified with liquid biopsy. *PLoS One*. 2018;13(12):e0209384. Published 2018 Dec 14. doi:10.1371/journal.pone.0209384
10	Zhang S, Zhu L, Xia B, et al. Epidermal growth factor receptor (EGFR) T790M mutation identified in plasma indicates failure sites and predicts clinical prognosis in non-small cell lung cancer progression during first-generation tyrosine kinase inhibitor therapy: a prospective observational study. *Cancer Commun (Lond)*. 2018;38(1):28. Published 2018 May 22. doi:10.1186/s40880-018-0303-2
11	Zhou Y, Ma Y, Shi H, Du Y, Huang Y. Epidermal growth factor receptor T790M mutations in non-small cell lung cancer (NSCLC) of Yunnan in southwestern China. *Sci Rep*. 2018;8(1):15426. Published 2018 Oct 18. doi:10.1038/s41598-018-33816-x
12	Hochmair MJ, Buder A, Schwab S, et al. Liquid-Biopsy-Based Identification of EGFR T790M

	Mutation-Mediated Resistance to Afatinib Treatment in Patients with Advanced EGFR Mutation-Positive NSCLC, and Subsequent Response to Osimertinib. *Target Oncol*. 2019;14(1):75-83. doi:10.1007/s11523-018-0612-z
13	Buder A, Setinek U, Hochmair MJ, et al. EGFR Mutations in Cell-free Plasma DNA from Patients with Advanced Lung Adenocarcinoma: Improved Detection by Droplet Digital PCR. *Target Oncol*. 2019;14(2):197-203. doi:10.1007/s11523-019-00623-x
14	Sundaresan TK, Sequist LV, Heymach JV, et al. Detection of T790M, the Acquired Resistance EGFR Mutation, by Tumor Biopsy versus Noninvasive Blood-Based Analyses. *Clin Cancer Res*. 2016;22(5):1103-1110. doi:10.1158/1078-0432.CCR-15-1031
15	Landi L, Tiseo M, Chiari R, et al. Activity of the EGFR-HER2 dual inhibitor afatinib in EGFR-mutant lung cancer patients with acquired resistance to reversible EGFR tyrosine kinase inhibitors. *Clin Lung Cancer*. 2014;15(6):411-417.e4. doi:10.1016/j.cllc.2014.07.002
16	Sakai K, Horiike A, Irwin DL, et al. Detection of epidermal growth factor receptor T790M mutation in plasma DNA from patients refractory to epidermal growth factor receptor tyrosine kinase inhibitor. *Cancer Sci*. 2013;104(9):1198-1204. doi:10.1111/cas.12211
**Incompatible study design (n=5)**	
#	Title
1	Reguart N, Rosell R, Cardenal F, et al. Phase I/II trial of vorinostat (SAHA) and erlotinib for non-small cell lung cancer (NSCLC) patients with epidermal growth factor receptor (EGFR) mutations after erlotinib progression. *Lung Cancer*. 2014;84(2):161-167. doi:10.1016/j.lungcan.2014.02.011
2	van der Wekken AJ, Kuiper JL, Saber A, et al. Overall survival in EGFR mutated non-small-cell lung cancer patients treated with afatinib after EGFR TKI and resistant mechanisms upon disease progression. *PLoS One*. 2017;12(8):e0182885. Published 2017 Aug 30. doi:10.1371/journal.pone.0182885
3	He J, Tan W, Tang X, Ma J. Variations in EGFR ctDNA Correlates to the Clinical Efficacy of Afatinib in Non Small Cell Lung Cancer with Acquired Resistance. *Pathol Oncol Res*. 2017;23(2):307-315. doi:10.1007/s12253-016-0097-y
4	Yanagita M, Redig AJ, Paweletz CP, et al. A Prospective Evaluation of Circulating Tumor Cells and Cell-Free DNA in EGFR-Mutant Non-Small Cell Lung Cancer Patients Treated with Erlotinib on a Phase II Trial. *Clin Cancer Res*. 2016;22(24):6010-6020. doi:10.1158/1078-0432.CCR-16-0909
5	Sacher AG, Paweletz C, Dahlberg SE, et al. Prospective Validation of Rapid Plasma Genotyping for the Detection of EGFR and KRAS Mutations in Advanced Lung Cancer [published correction appears in JAMA Oncol. 2016 Aug 1;2(8):1099]. *JAMA Oncol*. 2016;2(8):1014-1022. doi:10.1001/jamaoncol.2016.0173
**Insufficient data (n=4)**	
#	Title
1	Ke EE, Zhou Q, Zhang QY, et al. A Higher Proportion of the EGFR T790M Mutation May Contribute to the Better Survival of Patients with Exon 19 Deletions Compared with Those with L858R. *J Thorac Oncol*. 2017;12(9):1368-1375. doi:10.1016/j.jtho.2017.05.018
2	Jänne PA, Ou SI, Kim DW, et al. Dacomitinib as first-line treatment in patients with clinically or

	molecularly selected advanced non-small-cell lung cancer: a multicentre, open-label, phase 2 trial. *Lancet Oncol*. 2014;15(13):1433-1441. doi:10.1016/S1470-2045(14)70461-9
3	Lei L, Wang WX, Zhu YC, et al. Real-world efficacy and potential mechanism of resistance of icotinib in Asian advanced non-small cell lung cancer with EGFR uncommon mutations: A multi-center study. *Cancer Med*. 2020;9(1):12-18. doi:10.1002/cam4.2652
4	Hata A, Katakami N, Yoshioka H, et al. Spatiotemporal T790M Heterogeneity in Individual Patients with EGFR-Mutant Non-Small-Cell Lung Cancer after Acquired Resistance to EGFR-TKI. *J Thorac Oncol*. 2015;10(11):1553-1559. doi:10.1097/JTO.0000000000000647
**Without target outcome (n=2)**	
#	Title
1	Oda N, Hotta K, Ninomiya K, et al. A phase II trial of EGFR-TKI readministration with afatinib in advanced non-small-cell lung cancer harboring a sensitive non-T790M EGFR mutation: Okayama Lung Cancer Study Group trial 1403. *Cancer Chemother Pharmacol*. 2018;82(6):1031-1038. doi:10.1007/s00280-018-3694-5
2	Masago K, Fujita S, Hata A, et al. Validation of the digital PCR system in tyrosine kinase inhibitor-resistant EGFR mutant non-small-cell lung cancer. *Pathol Int*. 2018;68(3):167-173. doi:10.1111/pin.12630

Table S4. Patient characteristics in the retrieved studies

Author, year	Country	Stage	EGFR-TKI				Female, n (%)	Age, median (range), mean ± SD, y	Exon 19 deletion, n (%)	L858R mutation, n (%)	PFS, median (range), m	OS, median (range), m	Ref.
**Single-cohort study**													
Onitsuka 2010	Japan	IA-IV	G				7 (70.0)	61.5 (53-85)	5 (50.0)	5 (50.0)	7.0 (2.0-12.3)	21.1 (5.6-49.9)	43
Uramoto 2012	Japan	IA-IV	G				14 (73.7)	65.0 (52-87)	7 (36.8)	12 (63.2)	7.8 (2.0-34.7)	41.1 (4.5-118.9)	44
Ji 2013	Korea	N/A	G				16 (61.5)	58.0 (40-80)	16 (61.5)	10 (38.5)	11.2 (3.9-34.7)	33.8 (9.6-94.6)	45
Campo 2016	USA	advanced or recurrent				A	18 (75.0)	57 (27-83)	10 (41.7)	8 (33.3)	11.4 (95% CI: 5.9-13.7)	20.8 (95% CI: 15.1–40.5)	46
Liang 2017	Taiwan	IIIB-IV				A	87 (62.1)	61 (28–87)	84 (60.0)	27 (19.3)	N/A	N/A	47
Tanaka 2017	Japan	advanced or recurrent				A	15 (40.5)	65 (34-79)	21 (56.8)	13 (35.1)	6.8 (4.5-11.9)	N/A	48
**Multi-cohort study**													
Sequist 2011	USA	N/A	G	E			22 (59.5)	59.0 (37-88)	21 (56.8)	15 (40.5)	N/A	N/A	22
Yano 2011	Japan	N/A	G	E			14 (63.6)	59.5 (32-85)	11 (50.0)	9 (40.9)	10.85 (1.9-59.0)	N/A	23
Hata 2013	Japan	N/A	G	E			54 (69.2)	N/A	42 (53.8)	33 (42.3)	N/A	N/A	24
Sun 2013	Korea	advanced or recurrent	G	E			52 (74.3)	N/A	31 (44.3)	18 (25.7)	N/A	N/A	25
Li 2014	China	IV	G	E	I		25 (46.3)	51.2 (45.9-67.3)	53 (98.1)	0 (0.0)	13.0 (6.17-21.83)	31.6 (19.79-43.41)	26
Jin 2016	China	IV	G	E	I		47 (56.6)	61 (29-85)	25 (30.1)	25 (30.1)	N/A	N/A	27
Ko 2016	Japan	N/A	G	E		A	44 (72.1)	64 (39-84)	37 (60.7)	19 (31.1)	N/A	N/A	28
Matsuo 2016	Japan	advanced or recurrent	G	E		A	57 (78.1)	67 (48-82)	41 (56.2)	32 (43.8)	N/A	N/A	29
Nosaki 2016	Japan	advanced or metastatic	G	E		A	241 (61.0)	63 (27-84)	224 (56.7)	160 (40.5)	N/A	N/A	30
Takahama 2016	Japan	IIIB-IV	G	E		A	182 (70.0)	68 (36–90)	127 (48.8)	122 (46.9)	N/A	N/A	31
Tseng 2016	Taiwan	advanced	G	E		A	61 (62.2)	57.5 (30–83)	58 (59.2)	36 (36.7)	N/A	N/A	32
Lee 2017	Korea	IIIA-IV	G	E			12 (63.2)	58 (36-72)	14 (73.7)	3 (15.8)	7.5 (2.4-22.4)	N/A	33
Oya 2017	Japan	III-IV	G	E		A	110 (60.8)	65 (35-85)	97 (53.6)	81 (44.8)	10.5 (95% CI, 9.6-11.7)	N/A	34
Wang 2017	China	advanced or recurrent	G	E	I		53 (49.1)	57 (28–79)	70 (64.8)	33 (30.6)	12.3	32.8	35
Zhang 2017	China	IIIB-IV	G	E			32 (62.8)	58 (30-87)	31 (60.8)	20 (39.2)	2.0 (1.3–2.7)	6.8 (4.7–8.9)	36
Kaburagi 2018	Japan	III-IV	G	E		A	144 (61.8)	70 (32-93)	122 (52.4)	103 (44.2)	N/A	N/A	37
Lee 2019	Korea	N/A	G	E		A	52 (44.8)	55.8	26 (61.9)	13 (31.0)	N/A	N/A	38
Lin 2019	Taiwan	advanced or recurrent	G				98 (73.1)	71 (IQR: 60–80)	34 (82.9)	7 (17.1)	12.4 (9.8–15.0)	37.0 (95% CI, 25.1-40.9)	5
				E			46 (67.7)	67 (IQR: 61–73)	79 (68.1)	30 (25.9)	14.4 (10.8–18.0)	33.6 (95% CI not matured)	
						A	61 (61.6)	60 (IQR: 53–71)	48 (35.8)	76 (56.7)	12.4 (9.1–15.6)	not reached	
Yoon 2019	Korea	IIIB-IV	G				58 (47.2)	60.9 ± 11.5	27 (39.7)	37 (54.4)	11.5	29.3	39
						A	20 (48.8)	59.2 ± 12.3	59 (59.6)	23 (23.2)	13.4	28.5	
Dal Maso 2020	Italy	IIIB-IV	G	E		A	154 (65.5)	66 (33-92)	88 (71.5)	31 (25.2)	N/A	N/A	41
Del Re 2020	Italy	IIIB-IV	G	E			29 (69.1)	64.1 ± 8.6	27 (65.9)	11 (26.8)	10.2	N/A	40
						A	20 (48.8)	70.5 ± 11.3	127 (54.0)	87 (37.0)	14.4	N/A	
Wagener-Ryczek 2020	Germany	N/A	G	E		A	70 (56.9)	68 (40-87)	89 (72.4)	33 (26.8)	11.19 (6.19-24.15)	N/A	11
Oya 2021	Japan	III-IV	G	E		A	33 (53.2)	67 (36-80)	40 (64.5)	22 (35.5)	16.4	N/A	42

A: Afatinib; AR: acquired resistance; E: Erlotinib; EGFR-TKI: epidermal growth factor receptor-tyrosine kinase inhibitor; G: Gefitinib; I: Icotinib. The number of the references are the same as in the main text.

Table S5. P-score of the EGFR-TKIs in the network meta-analyses

Acquired T790M mutation rate					
All participants		Asian		Caucasian	
EGFR TKI	P-score	EGFR TKI	P-score	EGFR TKI	P-score
Erlotinib	0.8305	Gefitinib	0.8526	Erlotinib	0.9334
Gefitinib	0.7862	Erlotinib	0.7601	Gefitinib	0.3528
Afatinib	0.2049	Afatinib	0.1954	Afatinib	0.2138
Icotinib	0.1784	Icotinib	0.1919		

Table S6. Meta-regression model with univariate analysis of EGFR-TKIs

All participants					
Test for residual heterogeneity	QE (df = 58) = 172.7476, *p* value < 0.0001*				
Variance	Estimate	SE	*p* value	95% confidence intervals (CI)	
				Lower limit	Upper limit
Afatinib	0.3302	0.0341	< 0.0001*	0.2632	0.3971
Erlotinib	0.4692	0.0337	< 0.0001*	0.4032	0.5352
Gefitinib	0.4884	0.0273	< 0.0001*	0.4349	0.5418
Icotinib	0.3694	0.0824	< 0.0001*	0.2079	0.5310
Asian					
Test for residual heterogeneity	QE (df = 50) = 158.9904, *p* value < 0.0001*				
Variance	Estimate	SE	*p* value	95% confidence intervals (CI)	
				Lower limit	Upper limit
Afatinib	0.3002	0.0411	< 0.0001*	0.2197	0.3807
Erlotinib	0.4550	0.0372	< 0.0001*	0.3820	0.5279
Gefitinib	0.4899	0.0293	< 0.0001*	0.4324	0.5475
Icotinib	0.3692	0.0854	< 0.0001*	0.2018	0.5366
Caucasian					
Test for residual heterogeneity	QE (df = 5) = 3.2460, *p* value = 0.6621				
Variance	Estimate	SE	*p* value	95% confidence intervals (CI)	
				Lower limit	Upper limit
Afatinib	0.4219	0.0382	< 0.0001*	0.3470	0.4968
Erlotinib	0.5693	0.0572	< 0.0001*	0.4572	0.6813
Gefitinib	0.4884	0.0436	< 0.0001*	0.4029	0.5740

Table S7. Meta-regression model with univariate analysis of Asian and Caucasian

Gefitinib					
Test for residual heterogeneity	QE(df = 21) = 83.0444, *p* value < 0.0001*				
Variance	Estimate	SE	*p* value	95% confidence intervals (CI)	
				Lower limit	Upper limit
Asian	0.4898	0.0291	< 0.0001*	0.4328	0.5468
Caucasian	0.4750	0.1052	< 0.0001*	0.2688	0.6811
Erlotinib					
Test for residual heterogeneity	QE(df = 17) = 17.9175, *p* value = 0.3941				
Variance	Estimate	SE	*p* value	95% confidence intervals (CI)	
				Lower limit	Upper limit
Asian	0.4560	0.0228	< 0.0001*	0.4113	0.5006
Caucasian	0.5687	0.0591	< 0.0001*	0.4528	0.6846
Afatinib					
Test for residual heterogeneity	QE(df = 15) = 61.1539, *p* value < 0.0001*				
Variance	Estimate	SE	*p* value	95% confidence intervals (CI)	
				Lower limit	Upper limit
Asian	0.3007	0.0499	< 0.0001*	0.2028	0.3985
Caucasian	0.4118	0.0846	< 0.0001*	0.2460	0.5776

Table S8. Meta-regression model with univariate analysis of tissue and plasma biopsy

Gefitinib					
Test for residual heterogeneity	QE(df = 15) = 21.4786, *p* value = 0.1222				
Variance	Estimate	SE	*p* value	95% confidence intervals (CI)	
				Lower limit	Upper limit
Plasma	0.2732	0.0546	< 0.0001*	0.1662	0.3802
Tissue	0.5184	0.0218	< 0.0001*	0.4757	0.5611
Erlotinib					
Test for residual heterogeneity	QE(df = 11) = 14.0703, *p* value = 0.2291				
Variance	Estimate	SE	*p* value	95% confidence intervals (CI)	
				Lower limit	Upper limit
Plasma	0.4043	0.0880	< 0.0001*	0.2317	0.5768
Tissue	0.4609	0.0324	< 0.0001*	0.3975	0.5244
Afatinib					
Test for residual heterogeneity	QE(df = 9) = 16.0490, *p* value = 0.0659				
Variance	Estimate	SE	*p* value	95% confidence intervals (CI)	
				Lower limit	Upper limit
Plasma	0.1732	0.0771	0.0247*	0.0220	0.3244
Tissue	0.3808	0.0428	< 0.0001*	0.2969	0.4648

Table S9. ROBINS-I risk of bias assessment for multi-cohort studies of acquired T790M mutation rate

Author, year	Pre-intervention		At intervention	Post-intervention				Overall bias	Ref.
	Bias due to confounding	Bias in selection of participants into the study	Bias in classification of interventions	Bias due to deviations from intended interventions	Bias due to missing data	Bias in measurement of outcomes	Bias in selection of the reported result		
Sequist 2011	Moderate	Low	Low	Low	Low	Low	Low	***Moderate***	22
Yano 2011	Moderate	Low	Low	Low	Low	Low	Low	***Moderate***	23
Hata 2013	Moderate	Low	Low	Low	Low	Low	Low	***Moderate***	24
Sun 2013	Moderate	Low	Low	Low	Low	Low	Low	***Moderate***	25
Li 2014	Moderate	Low	Low	Low	Low	Low	Low	***Moderate***	26
Jin 2016	Low	Low	Low	Low	Low	Moderate	Low	***Low***	27
Ko 2016	Moderate	Low	Low	Low	Low	Moderate	Low	***Moderate***	28
Matsuo 2016	Moderate	Low	Low	Low	Low	Low	Low	***Moderate***	29
Nosaki 2016	Moderate	Low	Low	Low	Low	Low	Low	***Moderate***	30
Takahama 2016	Low	Low	Low	Low	Low	Low	Low	***Low***	31
Tseng 2016	Moderate	Low	Low	Low	Low	Moderate	Low	***Moderate***	32
Lee 2017	Moderate	Low	Low	Low	Low	Low	Low	***Moderate***	33
Oya 2017	Moderate	Low	Low	Low	Low	Low	Low	***Moderate***	34
Wang 2017	Moderate	Low	Low	Low	Low	Moderate	Low	***Moderate***	35
Zhang 2017	Moderate	Low	Low	Low	Low	Moderate	Low	***Moderate***	36
Kaburagi 2018	Moderate	Low	Low	Low	Low	Moderate	Low	***Moderate***	37
Lee 2019	Moderate	Low	Low	Low	Low	Low	Low	***Moderate***	38
Lin 2019	Moderate	Low	Low	Low	Low	Low	Low	***Moderate***	5
Yoon 2019	Moderate	Low	Low	Low	Low	Low	Low	***Moderate***	39
Dal Maso 2020	Moderate	Low	Low	Low	Low	Moderate	Low	***Moderate***	41
Del Re 2020	Moderate	Low	Low	Low	Low	Low	Low	***Moderate***	40
Wagener-Ryczek 2020	Moderate	Low	Low	Low	Low	Low	Low	***Moderate***	11
Oya 2021	Moderate	Low	Low	Low	Low	Moderate	Low	***Moderate***	42

The number of the references are the same as in the main text.

Figure S1. Acquired T790M mutation rate detected by tissue or plasma biopsy after treatment with (A) Gefitinib; (B) Erlotinib; (C) Afatinib.

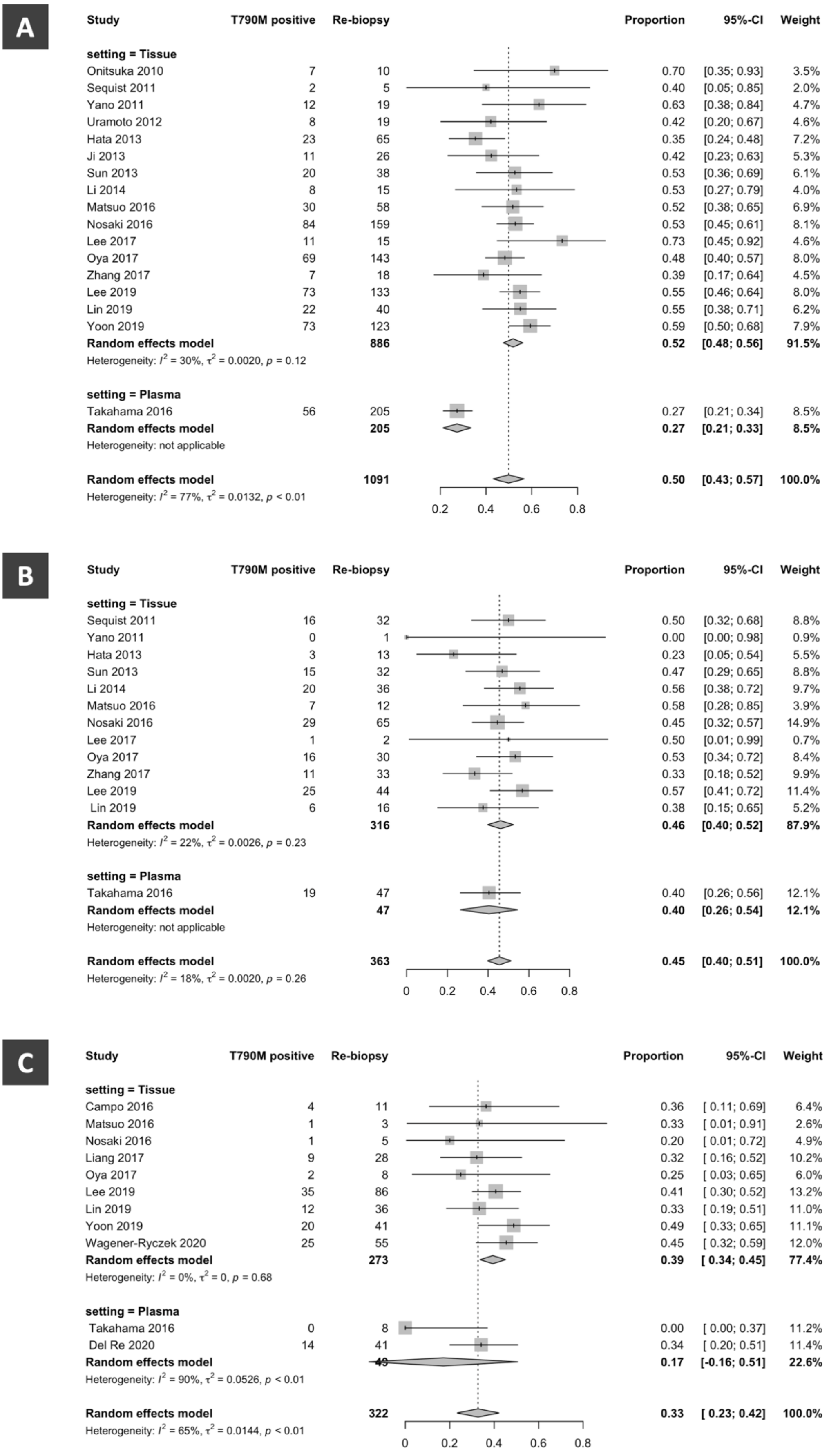

Figure S2. Funnel plots of the network meta-analysis of studies for (A) all participants ;(B) Asians.

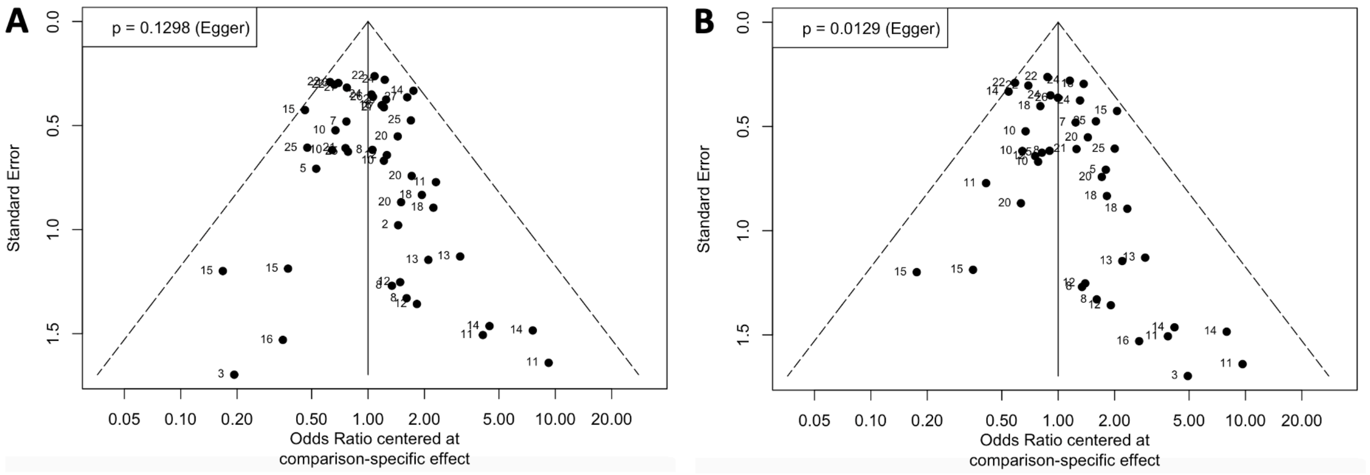

Figure S3. Funnel plots of the single-arm meta-analysis of studies for (A) all participants ;(C) Asians. Small study bias of the single-arm meta-analysis of studies for (B) all participants ;(D) Asians.

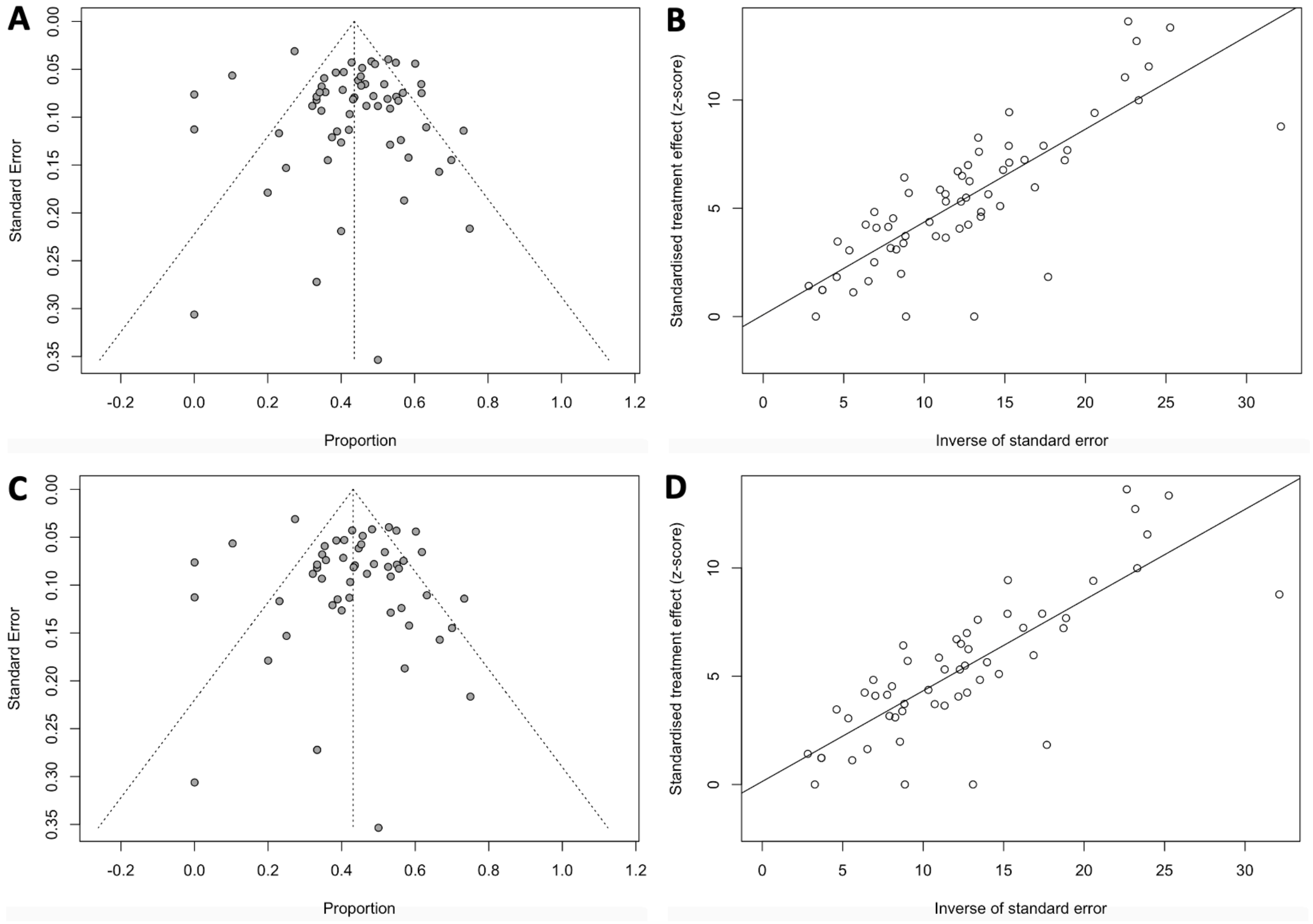

Figure S4. Direct evidence plots of the network meta-analysis for (A) all participants ;(B) Asians ;(C) Caucasian.

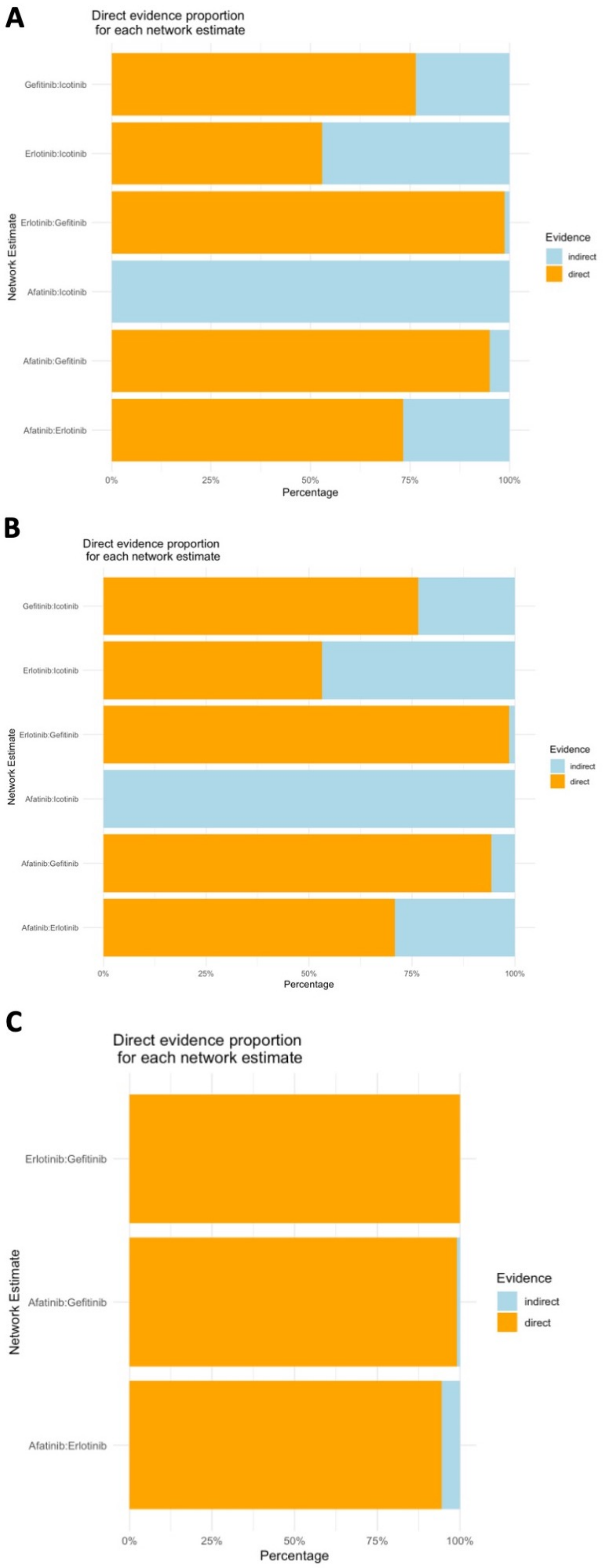

Figure S5. Node-splitting analysis of the network meta-analysis for (A) all participants ;(B) Asians ;(C) Caucasian.

A

```
> ## node-splitting model
> netsplit(net1)
Separate indirect from direct evidence (SIDE) using back-calculation method

Random effects model:

         comparison  k prop    nma direct indir.    RoR     z p-value
  Erlotinib:Afatinib 10 0.73 1.4773 1.5356 1.3285 1.1559  0.41  0.6793
  Gefitinib:Afatinib 11 0.95 1.4515 1.4437 1.6063 0.8988 -0.17  0.8644
   Icotinib:Afatinib  0    0 0.9120      . 0.9120      .     .       .
 Erlotinib:Gefitinib 19 0.99 1.0178 1.0363 0.2543 4.0752  1.48  0.1395
  Erlotinib:Icotinib  3 0.53 1.6197 1.8646 1.3827 1.3485  0.46  0.6445
  Gefitinib:Icotinib  3 0.76 1.5914 1.4228 2.2887 0.6217 -0.63  0.5274

Legend:
 comparison - Treatment comparison
 k          - Number of studies providing direct evidence
 prop       - Direct evidence proportion
 nma        - Estimated treatment effect (OR) in network meta-analysis
 direct     - Estimated treatment effect (OR) derived from direct evidence
 indir.     - Estimated treatment effect (OR) derived from indirect evidence
 RoR        - Ratio of Ratios (direct versus indirect)
 z          - z-value of test for disagreement (direct versus indirect)
 p-value    - p-value of test for disagreement (direct versus indirect)
```

B

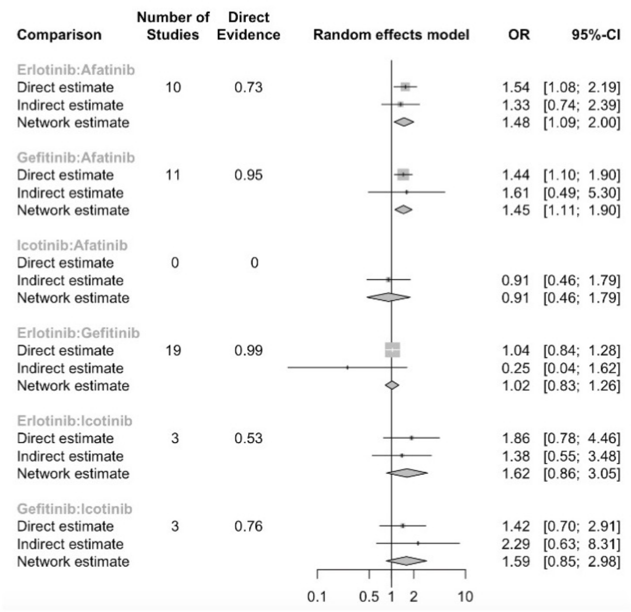

C

```
> ## node-splitting model
> netsplit(net1)
Separate indirect from direct evidence (SIDE) using back-calculation method

Random effects model:

         comparison  k prop    nma direct indir.    RoR     z p-value
  Erlotinib:Afatinib  9 0.71 1.4705 1.4658 1.4820 0.9891 -0.03  0.9765
  Gefitinib:Afatinib 10 0.94 1.5243 1.5373 1.3238 1.1613  0.23  0.8195
   Icotinib:Afatinib  0    0 0.9385      . 0.9385      .     .       .
 Erlotinib:Gefitinib 17 0.99 0.9647 0.9840 0.2416 4.0726  1.47  0.1413
  Erlotinib:Icotinib  3 0.53 1.5669 1.8646 1.2861 1.4497  0.57  0.5676
  Gefitinib:Icotinib  3 0.77 1.6242 1.4228 2.5038 0.5683 -0.75  0.4538

Legend:
 comparison - Treatment comparison
 k          - Number of studies providing direct evidence
 prop       - Direct evidence proportion
 nma        - Estimated treatment effect (OR) in network meta-analysis
 direct     - Estimated treatment effect (OR) derived from direct evidence
 indir.     - Estimated treatment effect (OR) derived from indirect evidence
 RoR        - Ratio of Ratios (direct versus indirect)
 z          - z-value of test for disagreement (direct versus indirect)
 p-value    - p-value of test for disagreement (direct versus indirect)
```

D

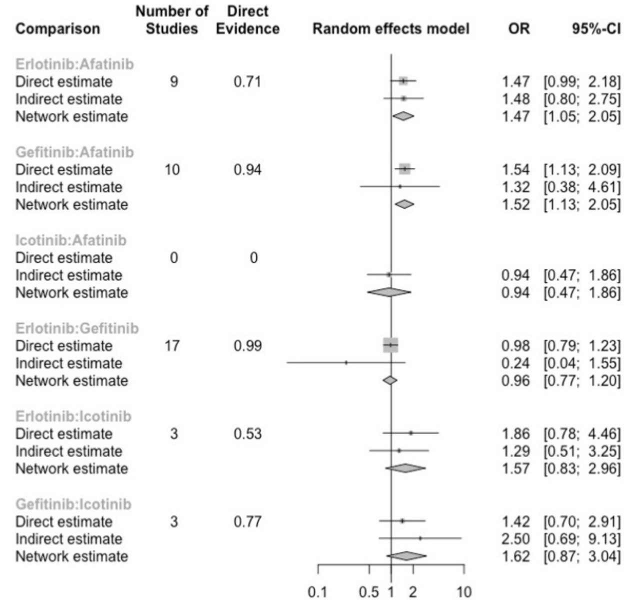

E

```
> ## node-splitting model
> netsplit(net1)
Separate indirect from direct evidence (SIDE) using back-calculation method

Random effects model:

          comparison k prop    nma direct indir.    RoR     z p-value
  Erlotinib:Afatinib 1 0.95 1.8465 1.8657 1.5452 1.2075  0.11  0.9148
  Gefitinib:Afatinib 1 0.99 1.1159 1.1123 1.5875 0.7006 -0.11  0.9148
 Erlotinib:Gefitinib 2 1.00 1.6548 1.6548      .      .     .       .

Legend:
 comparison - Treatment comparison
 k          - Number of studies providing direct evidence
 prop       - Direct evidence proportion
 nma        - Estimated treatment effect (OR) in network meta-analysis
 direct     - Estimated treatment effect (OR) derived from direct evidence
 indir.     - Estimated treatment effect (OR) derived from indirect evidence
 RoR        - Ratio of Ratios (direct versus indirect)
 z          - z-value of test for disagreement (direct versus indirect)
 p-value    - p-value of test for disagreement (direct versus indirect)
```

F

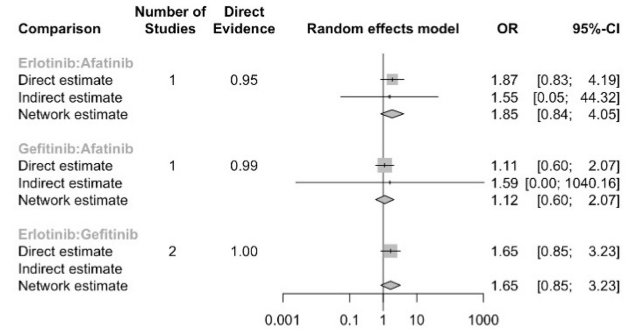

Figure S6. Design-by-treatment interaction model of the network meta-analysis for (A) all participants ;(B) Asians ;(C) Caucasian.

A

```
> ## Design-by-treatment interaction model
> decomp.design(net1)
Q statistics to assess homogeneity / consistency

                    Q df p-value
Total           26.60 30  0.6443
Within designs  25.04 27  0.5724
Between designs  1.56  3  0.6681

Design-specific decomposition of within-designs Q statistic

                       Design     Q df p-value
          Erlotinib:Gefitinib  1.54  5  0.9083
 Afatinib:Erlotinib:Gefitinib 21.67 18  0.2469
 Erlotinib:Gefitinib:Icotinib  1.82  4  0.7680

Between-designs Q statistic after detaching of single designs

              Detached design    Q df p-value
           Afatinib:Gefitinib 1.54  2  0.4641
          Erlotinib:Gefitinib 0.19  2  0.9097
 Afatinib:Erlotinib:Gefitinib 1.12  1  0.2908
 Erlotinib:Gefitinib:Icotinib 1.30  2  0.5222

Q statistic to assess consistency under the assumption of
a full design-by-treatment interaction random effects model

                   Q df p-value tau.within tau2.within
Between designs 1.56  3  0.6681          0           0
```

B

```
> ## Design-by-treatment interaction model
> decomp.design(net1)
Q statistics to assess homogeneity / consistency

                    Q df p-value
Total           24.05 27  0.6275
Within designs  22.35 24  0.5582
Between designs  1.70  3  0.6376

Design-specific decomposition of within-designs Q statistic

                       Design     Q df p-value
          Erlotinib:Gefitinib  0.94  4  0.9186
 Afatinib:Erlotinib:Gefitinib 19.59 16  0.2394
 Erlotinib:Gefitinib:Icotinib  1.82  4  0.7680

Between-designs Q statistic after detaching of single designs

              Detached design    Q df p-value
           Afatinib:Gefitinib 1.70  2  0.4281
          Erlotinib:Gefitinib 0.28  2  0.8704
 Afatinib:Erlotinib:Gefitinib 1.41  1  0.2355
 Erlotinib:Gefitinib:Icotinib 1.27  2  0.5301

Q statistic to assess consistency under the assumption of
a full design-by-treatment interaction random effects model

                   Q df p-value tau.within tau2.within
Between designs 1.70  3  0.6376          0           0
```

C

```
> ## Design-by-treatment interaction model
> decomp.design(net1)
Q statistics to assess homogeneity / consistency

                   Q df p-value
Total           0.01  1  0.9148
Within designs  0.00  0      --
Between designs 0.01  1  0.9148

Between-designs Q statistic after detaching of single designs

              Detached design    Q df p-value
          Erlotinib:Gefitinib 0.00  0      --
 Afatinib:Erlotinib:Gefitinib 0.00  0      --

Q statistic to assess consistency under the assumption of
a full design-by-treatment interaction random effects model

                   Q df p-value tau.within tau2.within
Between designs 0.01  1  0.9148          0           0
```

Figure S7. Influence analysis of the single-arm meta-analysis of Asians for (A) gefitinib ;(B) erlotinib ;(C) icotinib; (D) afatinib.

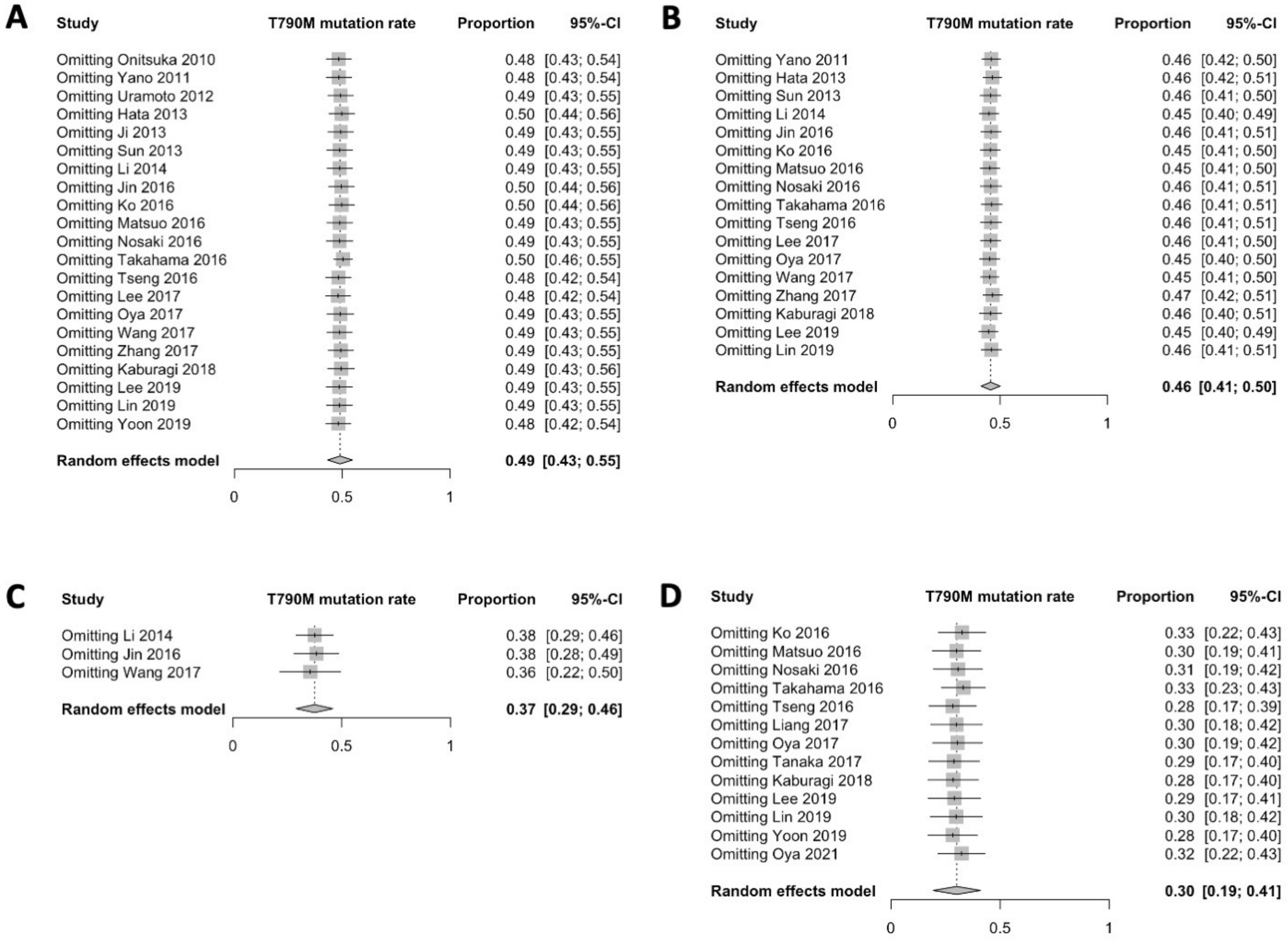

Figure S8. Influence analysis of the single-arm meta-analysis of Caucasians for afatinib.

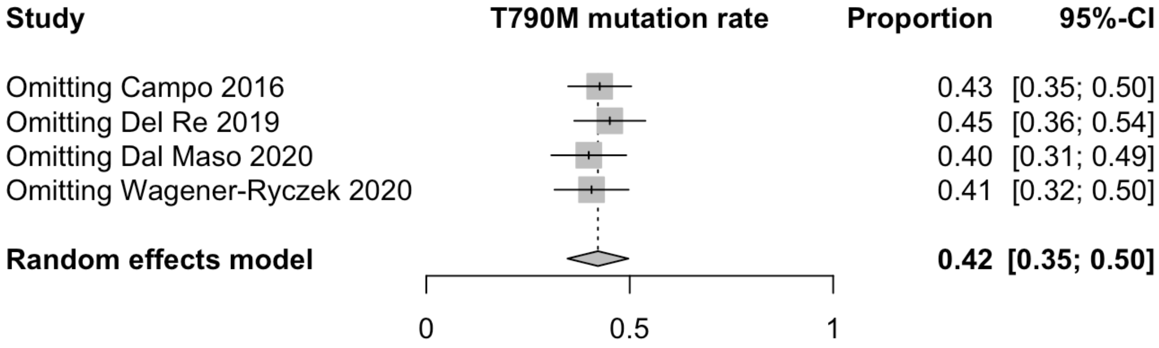

Supplement: Supplementary file 1 [file DataSheet_1.pdf]
